# PCSK9 Promotes the Malignancy of Triple‐negative Breast Cancer Cells by Reducing Cholesterol Levels at the Plasma Membrane to Activate EGFR and HER3

**DOI:** 10.1002/advs.202408514

**Published:** 2025-04-07

**Authors:** Tianhong Li, Renfei Wu, Kathy Qian Luo

**Affiliations:** ^1^ Department of Biomedical Sciences Faculty of Health Sciences University of Macau Taipa Macao SAR 999078 China; ^2^ Ministry of Education Frontiers Science Center for Precision Oncology University of Macau Taipa Macao SAR 999078 China

**Keywords:** EGFR, lipid raft, PCSK9, proliferation and metastasis, triple‐negative breast cancer

## Abstract

Triple‐negative breast cancer (TNBC) is a highly heterogeneous and clinically aggressive disease with the highest mortality rate among all subtypes of breast cancer. To discover new driver genes for metastatic TNBC, this work compares the transcription profiles of MDA‐MB‐231‐GFP cells and 231‐GFP‐derived lung metastatic cells (4–11). Results reveal that proprotein convertase subtilisin/kexin type 9 (PCSK9) is highly upregulated in 4–11 cells. Knockdown of PCSK9 greatly decreases the tumorigenic and metastatic potential of 4–11 cells, whereas overexpression of PCSK9 significantly enhances tumor maliganancy. Mechanistically, the binding of PCSK9 to the low‐density lipoprotein receptor (LDLR) results in decreased LDLR at the plasma membrane, which further decreases cholesterol and lipid raft in the plasma membrane and activates human epidermal growth factor receptor 1 and 3 (EGFR and HER3). Subsequently, phosphorylated EGFR and HER3 activate the Src/ERK/c‐Jun to increase the levels of cyclin D3 and vimentin and thereby enhance cell growth and metastasis. Metadata analyses also reveal that TNBC patients with high PCSK9 expression exhibited worse clinical outcomes. Taken together, these findings not only reveal a novel mechanism by which PCSK9 promotes the malignant potential of TNBC but also indicate that PCSK9 is a potential therapeutic target for treating TNBC patients.

## Graphical abstract

By decreasing cholesterol and lipid raft levels in the plasma membrane, proprotein convertase subtilisin/kexin type 9 (PCSK9) boosts human epidermal growth factor receptor 1 and 3 (EGFR and HER3) activation, driving tumor growth and metastasis in triple‐negative breast cancer (TNBC). This study reveals a novel lipid metabolism mechanism in cancer progression and positions PCSK9 as a promising therapeutic target for metastatic TNBC.

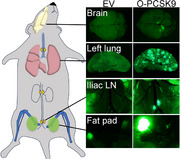

## 1 Introduction

Breast cancer is the most common cancer and the leading cause of cancer‐related fatalities among women globally. Triple‐negative breast cancer (TNBC) accounts for ≈10–15% of all breast cancers and is associated with significant psychological and treatment‐related burdens. TNBC is characterized by the absence of estrogen receptor (ER), progesterone receptor (PR), and human epidermal growth factor receptor 2 (HER2), as observed via immunohistochemistry (IHC) assays. This subtype has a poor prognosis and a high propensity for distant metastases.^[^ ^1^ ^]^ In contrast to non‐TNBC, which is commonly treated with endocrine and anti‐HER2 therapies, TNBC lacks effective targeted therapy. Although conventional chemotherapy remains the standard of care for many TNBC patients,^[^ ^2^, ^3^, ^4^ ^]^ treating early TNBC with a combination of chemotherapy and immunotherapy improved treatment outcomes.^[^ ^5^, ^6^ ^]^ Therefore, identifying novel driver genes for TNBC will provide new insights for designing more effective therapies to treat TNBC patients.

The human epidermal growth factor receptor (HER) family includes four receptor tyrosine kinases (RTKs), EGFR/HER1, HER2, HER3, and HER4, which activate the Ras–Raf–MEK–ERK signaling pathway to increase cell proliferation and the PI3K–AKT pathway to support cell survival, both of which are crucial for tumorigenesis.^[^ ^7^ ^]^ The activation or amplification of the epidermal growth factor receptor (EGFR) gene is frequently associated with various types of cancer.^[^ ^8^ ^]^ Up to 66% of basal‐like breast tumors and TNBCs exhibit highly activated EGFR signaling or EGFR amplification.^[^ ^9^, ^10^, ^11^ ^]^ However, anti‐EGFR monoclonal antibodies (e.g., cetuximab) have shown limited success against EGFR‐driven TNBC. Inhibitors such as cetuximab and panitumumab can suppress TNBC cell growth in vitro, but their clinical utilization has not been approved, possibly due to compensatory oncogenic pathways (such as activation or amplification of HER3) in vivo.^[^ ^12^, ^13^, ^14^ ^]^ Following therapy with EGFR‐targeted antibodies (panitumumab/cetuximab), some TNBC patients have been reported to exhibit residual tumors with increased HER3 expression and EGFR/HER3 dimerization—an activating interaction.^[^ ^15^ ^]^ In another report, the expression of HER3 itself reduced the efficacy of cetuximab by enhancing EGFR‐HER3 heterodimerization and downstream pathway activation.^[^ ^16^ ^]^ Additionally, high expression of both HER3 and EGFR could predict a poor prognosis in TNBC patients. Specifically, reports indicated that high expression of both HER3 and EGFR predicts a poor 10‐year prognosis after adjuvant chemotherapy, whereas low expression indicates a more favorable outcome.^[^ ^17^ ^]^ Indeed, targeted therapeutic strategies against HER3 may hold promise in treating TNBC.^[^ ^18^, ^19^, ^20^ ^]^ These results indicate the need to study the concomitant blockade of EGFR and HER3 in TNBC within a clinical setting.

Proprotein convertase subtilisin/kexin type 9 (PCSK9) is a serine protease involved in regulating cholesterol metabolism. Cholesterol can be synthesized within cells or taken up from outside cells. As cholesterol is not water soluble, they need to bind to low‐density lipoprotein (LDL) to form particles, which can then be endocytosed upon binding with LDL receptor (LDLR). Under normal conditions, PCSK9 binds to the LDL/LDLR complex and directs to intracellular, thus PCSK9 is crucial for maintaining cholesterol homeostasis.^[^ ^21^, ^22^ ^]^ More recently, elevated levels of PCSK9 have been observed in different types of cancer, including stomach, all breast (not mentioned for TNBC), thyroid, liver, and colon cancer.^[^ ^23^, ^24^, ^25^ ^]^ One mechanism involved in the promotion of tumorigenesis by PCSK9 is to decrease tumor cell apoptosis via the mitochondrial signaling pathway in hepatocellular carcinoma, neuroglioma and lung adenocarcinoma.^[^ ^26^, ^27^, ^28^ ^]^ In a separate discovery in 2020, Xinjian Liu reported that PCSK9 interacts with major histocompatibility complex (MHC) class I on the surface of tumor cells, facilitating its endocytosis to lysosomes and subsequent degradation.^[^ ^29^ ^]^ PCSK9 has been reported to promote melanoma oncogenesis by modulating the immune response.^[^ ^30^ ^]^ Although high expression of PCSK9 have been observed in several types of cancer, its oncogenic role in TNBC remains unclear.

In this study, we established a metastatic cell line, 4–11, from a commonly used TNBC cell line. Through RNA sequencing analysis, we demonstrated that overexpression of PCSK9 promoted the proliferation and metastasis of 4–11 cells. Gene knockdown and overexpression experiments further revealed the importance of PCSK9 in tumor growth and metastasis. We also elucidated the oncogenic mechanisms of PCSK9 and investigated the clinical relevance of its high expression with respect to the survival of TNBC patients.

## 2 Results

### 2.1 4–11 Cells Exhibit Greater Growth and Metastatic Potential than Parental 231‐GFP Cells

**Figure 1:**
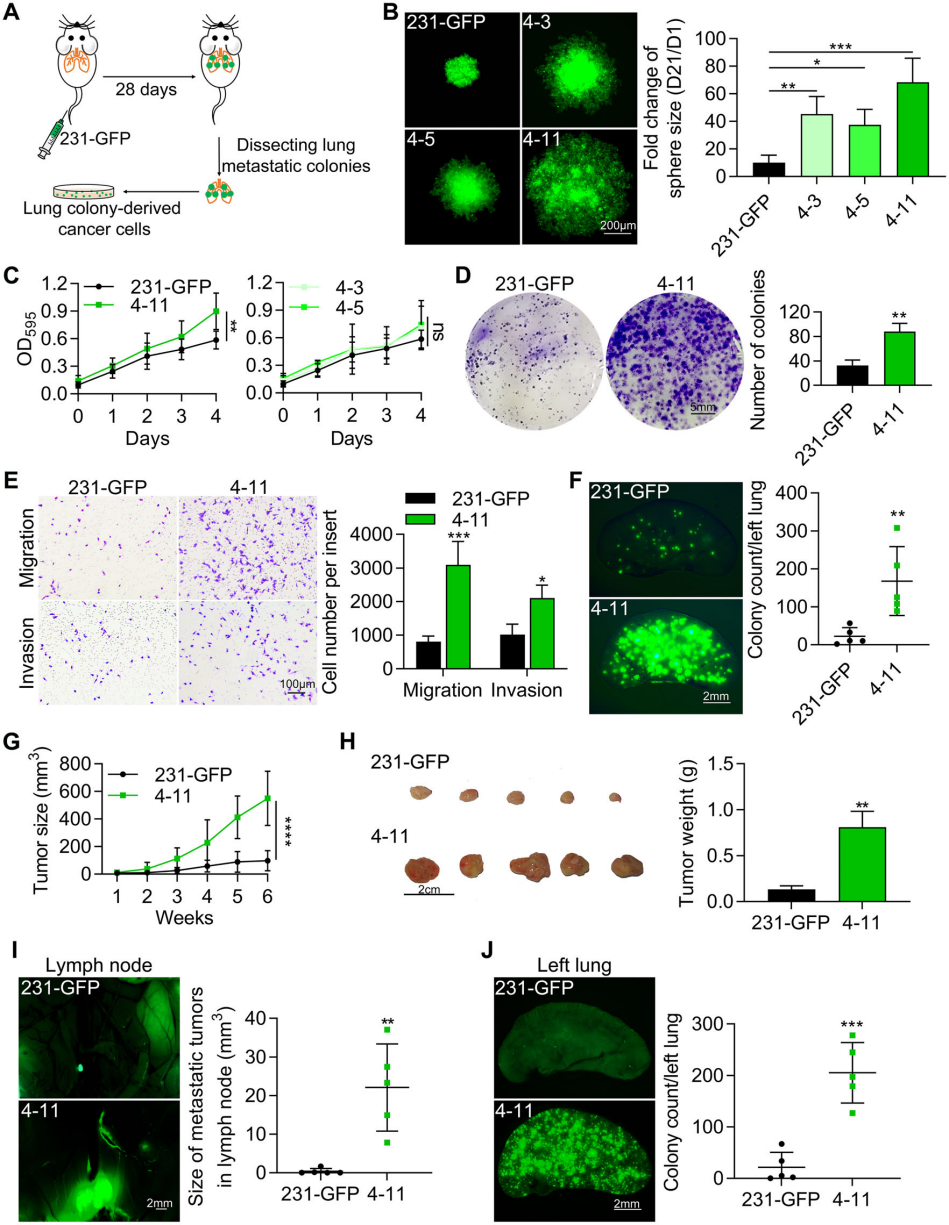
Analysis of the proliferative and metastatic capabilities of 231‐GFP cells. A) Schematics illustrating the generation of metastatic TNBC cell lines from parental MDA‐MB‐231‐GFP (231‐GFP) cells via the tail vein injection. B) Representative images and quantified results of tumor spheres in 3D culture. C) MTT assay was performed to assess the cell growth of the four cell lines. One thousand cells were cultured in 96‐well plates for 4 days. D) Representative images and quantified results of colony formation assay of 231‐GFP and 4–11 cells with 1000 cells cultured in 6‐well plates for 12 days. E) Representative images and quantified results of migrated cells in transwell migration and invasion assays. 1 × 10^4^ cells were cultured for 16 h in the migration assays. 2 × 10^4^ cells were cultured for 24 h in the invasion assays. F) Representative images and quantified results of lung colony formation assay in vivo (*n* = 5). G) Primary tumors were monitored weekly for 6 weeks (*n* = 5). H) Representative images and quantified results of primary tumors. I,J) Representative images and quantified results of metastatic tumors in lymph nodes (I) or left lungs (J). The data are presented as the means ± SD. The significance of differences was determined by one‐way ANOVA (B), two‐way ANOVA (C, E, G) or Student’s *t‐test* (D, F, H, I, J) (*p < 0.05, **p < 0.01, ***p < 0.001, ****p < 0.0001 and ns, not significant).

To initiate our studies, we intravenously injected parental MDA‐MB‐231‐GFP (231‐GFP) TNBC cells into nude mice, isolated lung metastatic cells, and cultured several clonal cell lines (Figure **1**A).^[^ ^31^ ^]^ Each clone was uniquely labeled according to the from which it was derived and the number of clones (e.g., 4–11, indicating the 11th tumor from the lung of the fourth mouse). Three cell lines (4–3, 4–5, and 4–11) derived from the fourth mouse were selected for further analysis. We used 3D tumor sphere formation and 2D cell culture‐based MTT assays to assess their proliferative abilities. Fluorescence images illustrated the formation of larger spheres in the three cell lines than the parental 231‐GFP cells when cultured in 96‐well round bottom non‐attached plates coated with a hydrogel layer (Figure 1B). After a 21‐day culture period of 200 cells, we assessed the growth rate of spheres with day 1 as the reference point (Figure S1A, Supporting Information). The quantified results demonstrated that the sphere size of 4–11 cells increased 68.3‐fold, while the sphere sizes of 231‐GFP, 4–3 and 4–5 cells increased 10.1‐fold, 45.3‐fold, and 37.5‐fold, respectively (Figure 1B). Further analysis by MTT assay demonstrated that compared with 231‐GFP cells, 4–11 cells exhibited a relatively high proliferation rate (1.5‐fold) compared to the growth rate of 231‐GFP under 2D conditions in normal 96‐well plates (Figure 1C). The growth rates of the 4–3 and 4–5 cells demonstrated very similar, but not significantly different from the growth rate of the 231‐GFP cells. Moreover, the number of colonies formed by 4–11 cells was 2.7‐fold greater than that formed by 231‐GFP cells according to the colony formation assay (Figure 1D).

To further explore the migration and invasion potential of 4–11 cells in vitro, we performed transwell migration and matrigel‐coated transwell invasion assays. The results revealed a 3.8‐fold increase in the number of migrated cells compared to that in 231‐GFP cells (Figure 1E). Additionally, the number of invading cells was 1.9‐fold greater than that of 231‐GFP cells (Figure 1E).

Next, we assessed the colony formation potential of 4–11 cells in vivo by performing lung colony formation assay. Half a million cells were injected into the tail vein of nude mice, and the left lungs were taken photos after 28 days to determine the number of colonies. The results indicated that the lung colony counts of the 4–11 group were 5.6‐fold greater than that of the 231‐GFP cell group (Figure 1F).

To further explore the primary tumorigenic potential and spontaneous metastatic potential of 4–11 cells, we used an orthotopic model in which two million cancer cells were injected into the mammary fat pads of NOD/SCID mice. After 6 weeks, we observed the development of orthotopic tumors and metastatic tissues (Figure 1G–J). Weekly measurements of body weights revealed no significant differences between the 231‐GFP group and the 4–11 group (Figure S1B, Supporting Information). However, 4–11 cells formed significantly larger primary tumors, with 11.7‐fold and 7.4‐fold increases in tumor size and weight, respectively (Figure 1G,H). We observed a 59.4‐fold increase in the metastatic tumor size in the lymph node (Figure 1I) and a 9.5‐fold increase in the metastatic tumor counts in the left lung (Figure 1J).

In conclusion, our comprehensive assessments, including sphere formation, MTT, colony formation, transwell migration, lung colony formation, and orthotopic model assays, collectively established that compared with 231‐GFP cells, 4–11 cells exhibit significantly greater potential for both proliferation and metastasis.

### 2.2 4–11 and TNBC Cells Expressed Much Higher Levels of PCSK9

To determine why the 4–11 cell line outperform parental 231‐GFP cell line, we conducted RNA‐seq analysis to compare the gene expression profiles between the two cell lines. Genes, exhibiting fold change ≥ 2 or ≤ 0.5 and *p* < 0.05, were classified as differentially expressed genes. Genes were selected for detectability with fragments per kilobase of exon per million mapped fragments (FPKM) values exceed 2.

**Figure 2:**
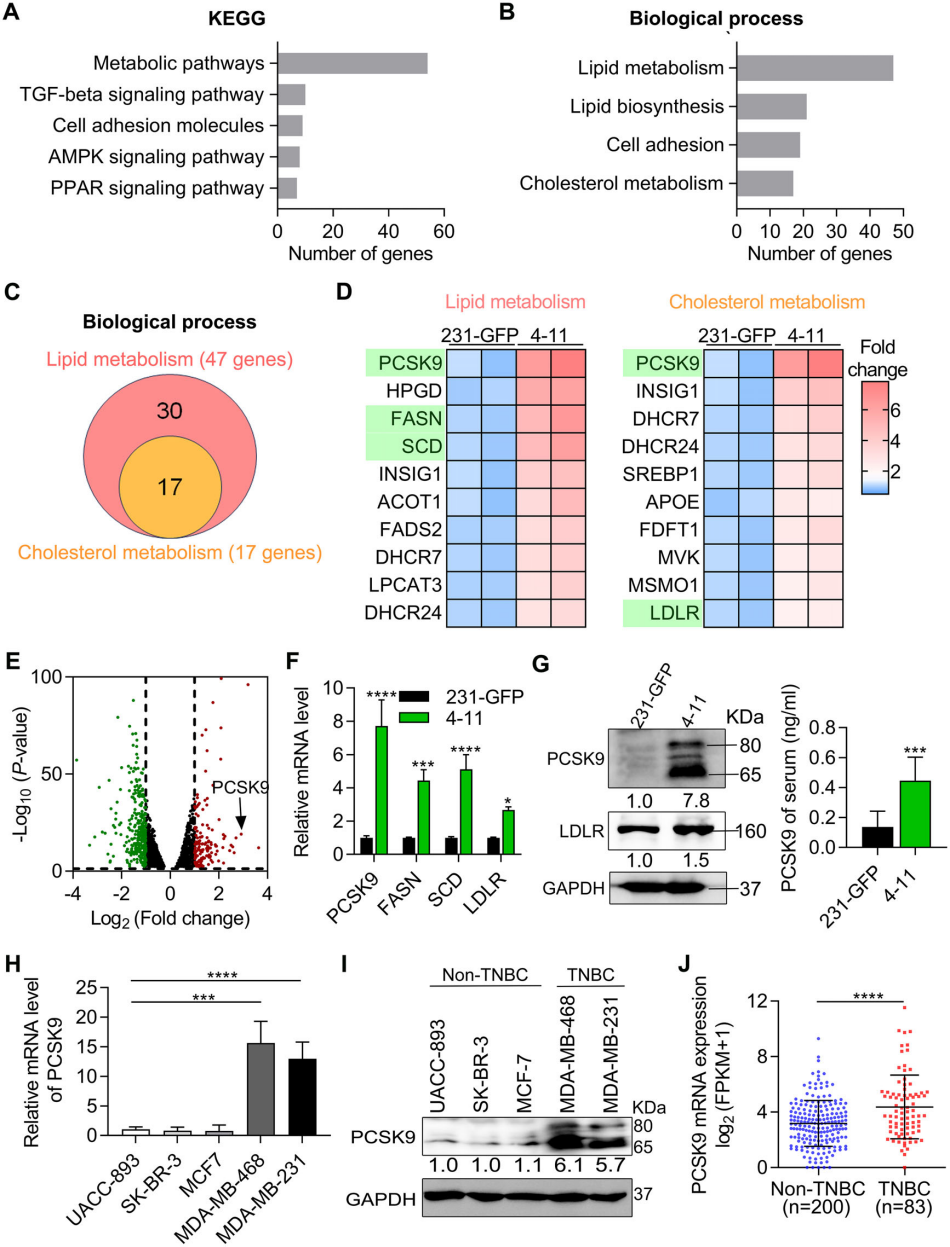
PCSK9 was highly expressed in 4–11 and TNBC cells. A) Kyoto Encyclopedia of Genes and Genomes (KEGG) enrichment analysis revealed pathways with the most significant differences from the transcriptomes of 4–11 cells and 231‐GFP cells. B) Functional enrichment analysis of biological process via the DAVID website revealed the metabolic pathways of 4–11 cells. C) Venn diagrams of differentially expressed genes (DEGs) in metabolic pathways. D) Heatmap displays the top ten genes in lipid metabolism and cholesterol metabolism pathways based on the RNA‐seq results (*p* < 0.05). E) Volcano plot depicted the upregulated (red dots) and downregulated (green dots) genes in 4–11 cells than 231‐GFP cells. (*p* < 0.05; fold change ≥2 or ≤ 0.5; FPKM of 231‐GFP ≥5) F) The qPCR results showed the mRNA levels of PCSK9, FASN, SCD and LDLR in 4–11 and 231‐GFP cells. G) Western blotting (left) showed the protein levels of PCSK9 and LDLR in 4–11 and 231‐GFP cells; ELISA assay (right) result showed the PCSK9 level in the serum of mice with orthotopic tumors injected with 231‐GFP and 4–11 cells (*n* = 5). H,I) The qPCR and Western blot results showed the mRNA (H) or protein (I) levels of PCSK9 in non‐TNBC and TNBC cell lines. J) The mRNA levels of PCSK9 between non‐TNBC and TNBC patients from RNA‐seq datasets in cBioPortal platform. The significance of differences was determined by one‐way ANOVA (H), two‐way ANOVA (F) or Student’s *t‐test* (G, J).

Utilizing KEGG enrichment analysis, among the 462 upregulated genes, we identified 54 genes associated with metabolic pathways and these pathways were related to hallmarks of cancer in terms of both proliferation and metastasis (Figure **2**A).^[^ ^32^ ^]^ Furthermore, functional enrichment analysis of biological process via the DAVID website revealed that 47 genes were linked to lipid metabolism (Figure 2B). Other pathways related to metabolism were also enriched, with 17 genes concurrently implicated in both lipid metabolism and cholesterol metabolism (Figure 2C).

To identify promising candidates for further exploration, we selected the top ten genes on lipid metabolism and cholesterol metabolism, individually (Figure 2D). Remarkably, proprotein convertase subtilisin/Kexin type 9 (PCSK9) emerged as the top upregulated in both the lipid metabolism and cholesterol metabolism pathways. With the FPKM set to greater than 5, PCSK9 ranked third among all upregulated mRNAs (Figure 2E). By quantitative polymerase chain reaction (qPCR) analysis, we assessed the mRNA levels, including the top upregulated PCSK9, SCD (stearoyl‐CoA desaturase) and FASN (fatty acid synthase) involved in de novo lipogenesis in lipid metabolism, and low‐density lipoprotein receptor (LDLR), which is targeted by PCSK9 for degradation. The mRNA levels were elevated in 4–11 cells compared to 231‐GFP cells, among which PCSK9 exhibited greatest increase of 7.7‐fold (Figure 2F). We further confirmed that the protein levels of PCSK9 also exhibited a substantial increase of 7.8‐fold in 4–11 cells versus the control 231‐GFP cells and the protein level of LDLR was 1.5‐fold higher in 4–11 cells than 231‐GFP cells (Figure 2G, left).

We then injected two million 231‐GFP or 4–11 cells into the mammary fat pads of NOD/SCID mice and let these cancer cells grow into primary tumors in 6 weeks, Afterward, we used ELISA assay to measure the protein levels of PCSK9 in the serum of the mice. The results showed that mice bearing 4–11 tumors secreted 3.3‐fold more PSCK9 proteins into the bloodstream than mice bearing 231‐GFP tumors (Figure 2G, right).

We further compared the levels of PCSK9 between two TNBC cell lines (MDA‐MB‐231 and MDA‐MB‐468) and three non‐TNBC breast cancer cell lines (UACC‐893, SK‐BR‐3, and MCF7) by qPCR and Western blotting analyses. The results showed that the two TNBC cell lines exhibited significantly higher mRNA levels of 13.0–15.6‐fold and protein levels of 5.7–6.1‐fold compared to those non‐TNBC cell line (Figure 2H,I). Analysis from the ARCHS4 website (website information added into Materials and Methods section) also confirmed these results (Figure S2A, Supporting Information). Furthermore, in the clinical dataset, we observed elevated expression of PCSK9 in 83 TNBC patients than 200 non‐TNBC patients (Figure 2J).^[^ ^33^ ^]^ Additionally, according to the TCGA dataset in the UALCAN platform, the average mRNA expression level of PCSK9 from 116 TNBC patients is significantly higher than 566 patients with a luminal type of breast cancer or 114 normal tissues from breast cancer patients (Figure S2B, Supporting Information).

### 2.3 Knockdown of PCSK9 Suppressed the Proliferation and Metastasis of 4–11 Cells

To investigate whether PCSK9 is responsible for the proliferation and metastasis of 4–11 cells, we used two short hairpin RNAs (shPCSK9‐1 and shPCSK9‐2) to knock down the *pcsk9* gene. The knockdown efficiencies were evaluated by qPCR and Western blotting (Figure S3A, Supporting Information).

**Figure 3:**
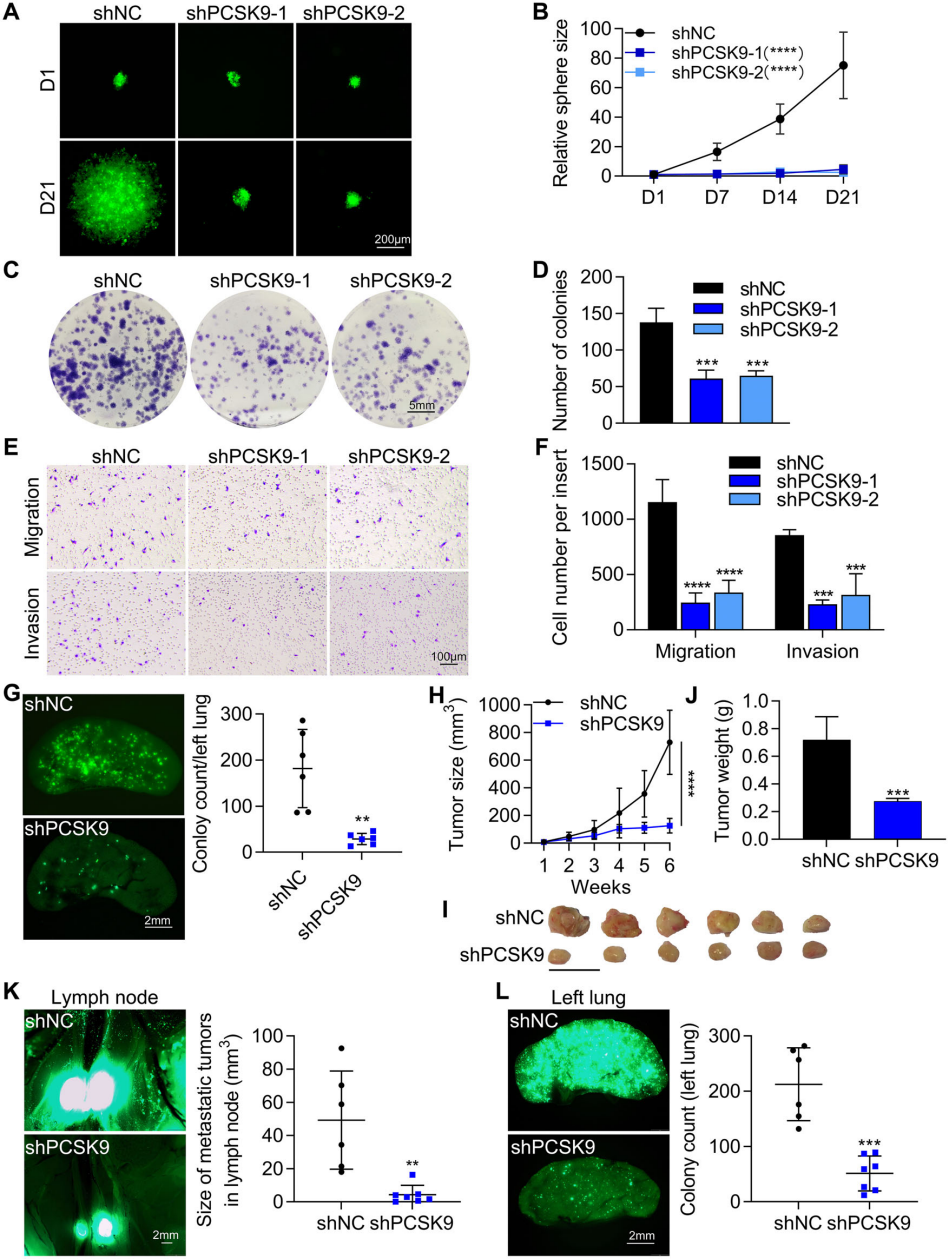
PCSK9 knockdown suppressed the proliferation and metastasis of 4–11 cells. A) Representative images of tumor sphere formation by 200 cultured cells. B) The quantified results displayed the ratio of sphere sizes every 7 days relative to day 1. C,D) Representative images (C) and quantified results (D) of colony formation assay of 4–11 cells after *pcsk9* knockdown. 1000 cells were cultured in 6‐well plates for 12 days. E,F) Representative images and quantified results of transwell migration and invasion assays in 4–11 cells after PCSK9 knockdown. Five thousand cells were cultured for 16 h in migration assays, ten thousand cells were cultured for 24 h in invasion assays. G) Representative images and quantified results of left lung colonies (*n* = 6). H) Primary tumor growth rates of shNC and shPCSK9 cells (*n* = 6–7). I,J) Representative images and quantified results of primary tumors formed by shNC and shPCSK9 cells after 6 weeks. K,L) Representative images and quantified results of metastatic tumors in lymph nodes (K) or left lungs (L). The significance of differences was determined by one‐way ANOVA (D), two‐way ANOVA (B, F, H), or Student’s *t‐test* (G, J, K, L).

Next, we confirmed the proliferative ability of *pcsk9*‐knockdown cells by performing sphere formation and colony formation assays in vitro. Following the transfection of 4–11 cells with shRNAs, the negative control (shNC) cells exhibited a consistently rapid sphere growth rate, while the two shRNAs targeting the *pcsk9* gene resulted in a significantly reduced sphere growth rate (Figure **3**A,B). The spheres of shNC cells were 21‐fold larger than two shPCSK9 cells. The results of colony formation assay also revealed a significant decrease in the number of colonies formed in two shPCSK9 than shNC cells (Figure 3C). The quantified results revealed that the colony count in the knockdown groups decreased by ≈50% compared to the shNC group (Figure 3D).

To further investigate the migration and invasion abilities in vitro, transwell assays were performed. The results indicated a significant decrease in the number of migrated and invaded cells compared with those in the shNC group (Figure 3E,F). Next, we examined the effect of PCSK9 knockdown on lung colony formation potential in vivo. Half a million shPCSK9‐1 (shPCSK9) cells were injected into the tail vein of nude mice, and the left lungs were photographed after 28 days to determine the number of colonies. Compared with the shNC group, fluorescence images revealed a significant reduction in the number of lung colonies in the shPCSK9 group, indicating that the degree of lung metastasis decreased by more than 90% (Figure 3G).

We further assessed the primary tumorigenic potential and spontaneous metastatic potential of shPCSK9 cells and injected two million cells into the mammary fat pads of NOD/SCID mice for 6 weeks (Figure 3H–L). No significant differences in body weight were detected between the knockdown group and the shNC group (Figure S3B, Supporting Information). The images indicated that the shPCSK9 group formed 0.17‐fold smaller primary orthotopic tumors, and the shPCSK9 group also weighed 66% less than the shNC group (Figure 3H–J). The results revealed that shPCSK9 cells formed much smaller metastatic tumors in the lymph nodes (a decrease in tumor size of ≈91%) (Figure 3K). More importantly, we observed a 76% reduction in the number of metastatic tumors in the left lung (Figure 3L).

Overall, we can conclude that the expression of PCSK9 is needed to support the proliferative and metastatic abilities of 4–11 cells according to the results of sphere formation, colony formation, transwell, lung metastasis and orthotopic model assays.

### 2.4 Overexpression of PCSK9 Promoted the Proliferation and Metastasis of TNBC Cells

To further confirm the importance of PCSK9 in promoting proliferation and metastasis in TNBC cells, we overexpressed the *pcsk9* gene in two TNBC MDA‐MB‐231‐GFP (231‐GFP) and MDA‐MB‐468‐Clover (468‐Clover) cell lines, as confirmed by qPCR and Western blotting (Figure S4A, Supporting Information).

**Figure 4:**
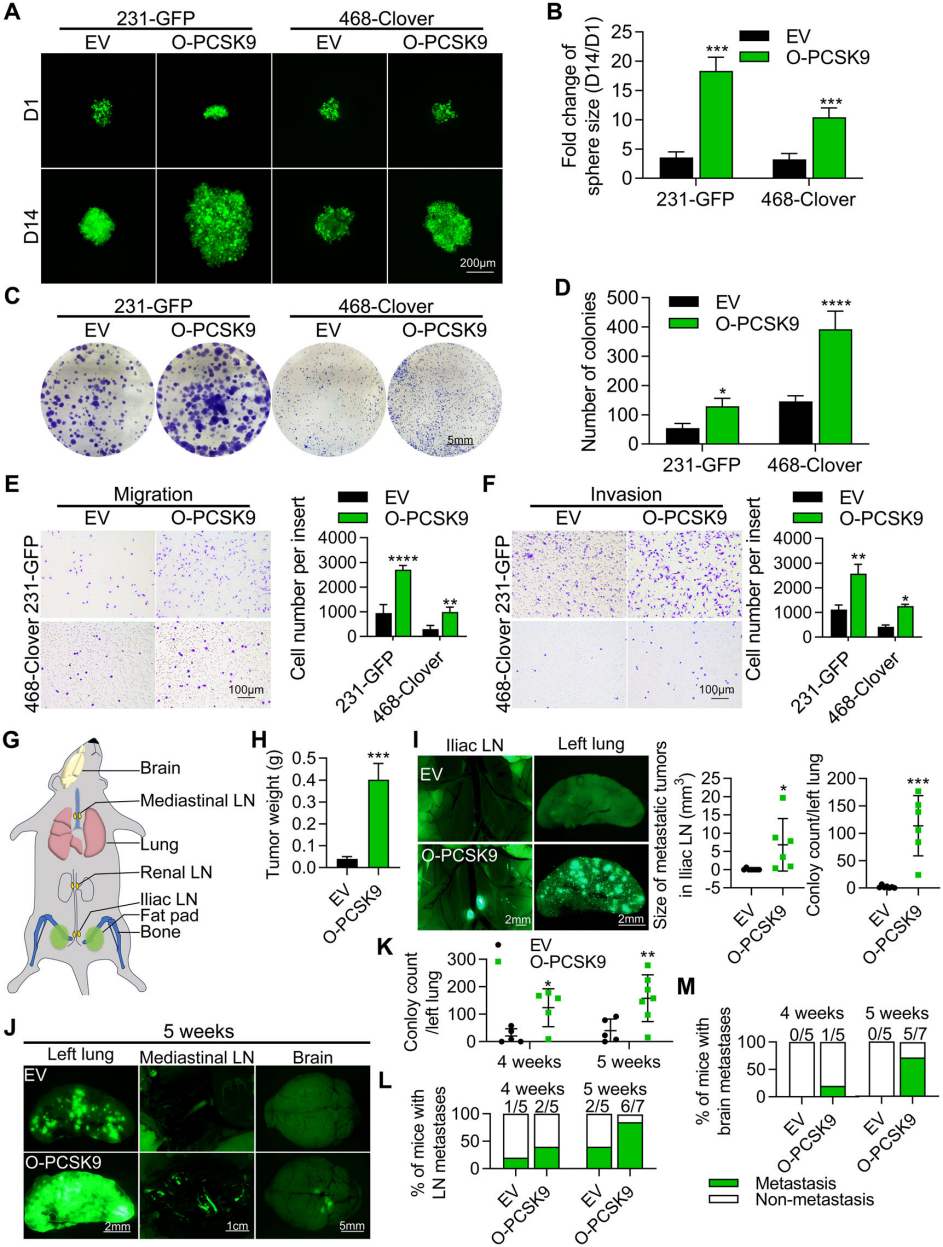
The overexpression of PCSK9 promoted the proliferation and metastasis of 231‐GFP and 468‐Clover cells. A,B) Representative images and quantified results of sphere formation assay of 231‐GFP and 468‐Clover cells transfected with PCSK9 overexpression plasmid (O‐PCSK9) or empty vector (EV). Two thousand cells were seeded in 96‐well round bottom plates. C,D) Representative images and quantified results of colony formation assay. One thousand 231‐GFP cells were cultured in 6‐well plates for 12 days. Five thousand 468‐Clover cells were cultured in 6‐well plates for 16 days. E,F) Representative images and quantified results of transwell migration (E) or invasion (F) assays. Ten thousand cells were cultured for 16 h in migration assays, and twenty thousand cells were cultured for 24 h in invasion assays. G) Schematics illustrating the parts of primary and metastatic tumors. H) The weight of primary tumors after 6 weeks. I) Representative images and quantified results of metastatic tumors in the iliac lymph node (LN) and left lungs. J) Representative images of left lung colonies and metastatic tumors after 5 weeks of tail vein injection (*n* = 5–7). K) Quantified results of left lung colonies after 4 and 5 weeks. L) Quantified results of metastatic tumors in mediastinal LN after 4 and 5 weeks. (M) Quantified results of metastatic tumors in brain after 4 and 5 weeks. The significance of differences was determined by two‐way ANOVA (B, D, E, F, K) or Student’s *t‐test* (H, I).

Fluorescence images revealed that overexpressing of PCSK9 (O‐PCSK9) formed larger spheres in the two cell lines (Figure **4**A). The quantified results demonstrated a substantial increase in sphere size by an 18.3‐fold increase in 231‐GFP cells and a 10.4‐fold increase in 468‐Clover cells with O‐PCSK9 compared to 1 day (Figure 4B). This high rate of growth stands out when compared to that of cells transfected with an empty vector (EV) (Figure 4B). Additionally, we detected an increase in colony formation ability in 231‐GFP and 468‐Clover cells with O‐PCSK9, resulting in increases of 2.5‐fold and 8.3‐fold, respectively, compared to the EV control cells (Figure 4C,D). Moreover, O‐PCSK9 significantly elevated the migration and invasion abilities of 231‐GFP and 468‐Clover cells by 2.3‐3.4‐fold (Figure 4E,F).

We further investigated the effects of overexpressing PCSK9 on tumor growth and metastasis in 231‐GFP cells in vivo. Two million 231‐GFP cells overexpressing PCSK9 or an empty vector were injected into the mammary fat pads of NOD/SCID mice and within the 6 weeks period, we examined the development of orthotopic and metastatic tumors (Figure 4G, Figure S4E, Supporting Information). Although there were no significant differences in the body weight between the O‐PCSK9 group and the EV group (Figure S4B, Supporting Information), overexpressing PCSK9 greatly increased the primary tumor weight by over 10‐fold and tumor size by ≈4.9‐fold compared to the empty vector group (Figure 4H, Figure S4C,D, Supporting Information). In addition, we also assessed the metastatic potential of overexpressing PCSK9 in 231‐GFP cells. The imaging analyses showed that overexpressing PCSK9 significantly enhanced the ability of 231‐GFP cells to metastasize from the primary tumor in the mammary fat pad to the nearby iliac lymph node (LN) with tumor size being increased by 71‐fold and the metastatic lung colony numbers being enhanced by 57‐fold (Figure 4I). We further confirmed these findings by immunohistochemistry (IHC) on the left lung samples and observed more tumors in the O‐PCSK9 group than in the EV group (Figure S4F, Supporting Information).

During this animal experiment, we did not observe the formation of metastatic tumors in the other parts of the animal including the bone, renal lymph node (LN), mediastinal LN, intestine, liver, heart, and the brain (Figure S4E, Supporting Information). To further investigate secondary metastatic sites, half a million 231‐GFP cells overexpressing PCSK9 were injected into the tail vein of nude mice, and metastatic progression was monitored over 1 day, 1 week, 2 weeks, 4 weeks, and 5 weeks. Fluorescence imaging revealed the development of metastatic tumors in the mediastinal LN and brain in the O‐PCSK9 group after 4 and 5 weeks (Figure 4J, Figure S4G, Supporting Information). Notably, the number of colonies in the left lung increased by 5.9‐fold and 3.9‐fold at 4‐ and 5‐week post‐injections, respectively (Figure 4K). The quantified results showed significant metastases in the nearby mediastinal LN and distant brain, with ≈85.7% and 71.4% of the O‐PCSK9 group exhibiting metastases in the distant lymph node and the brain after 5 weeks of intravenous injection (Figure 4L,M). Overall, we can conclude that PCSK9 plays an important role in increasing the proliferation and metastasis of TNBC cells in vitro and in vivo.

### 2.5 PCSK9 Activated EGFR/HER3‐ERK‐cJun Signaling to Promote the Proliferation and Metastasis of TNBC Cells

**Figure 5:**
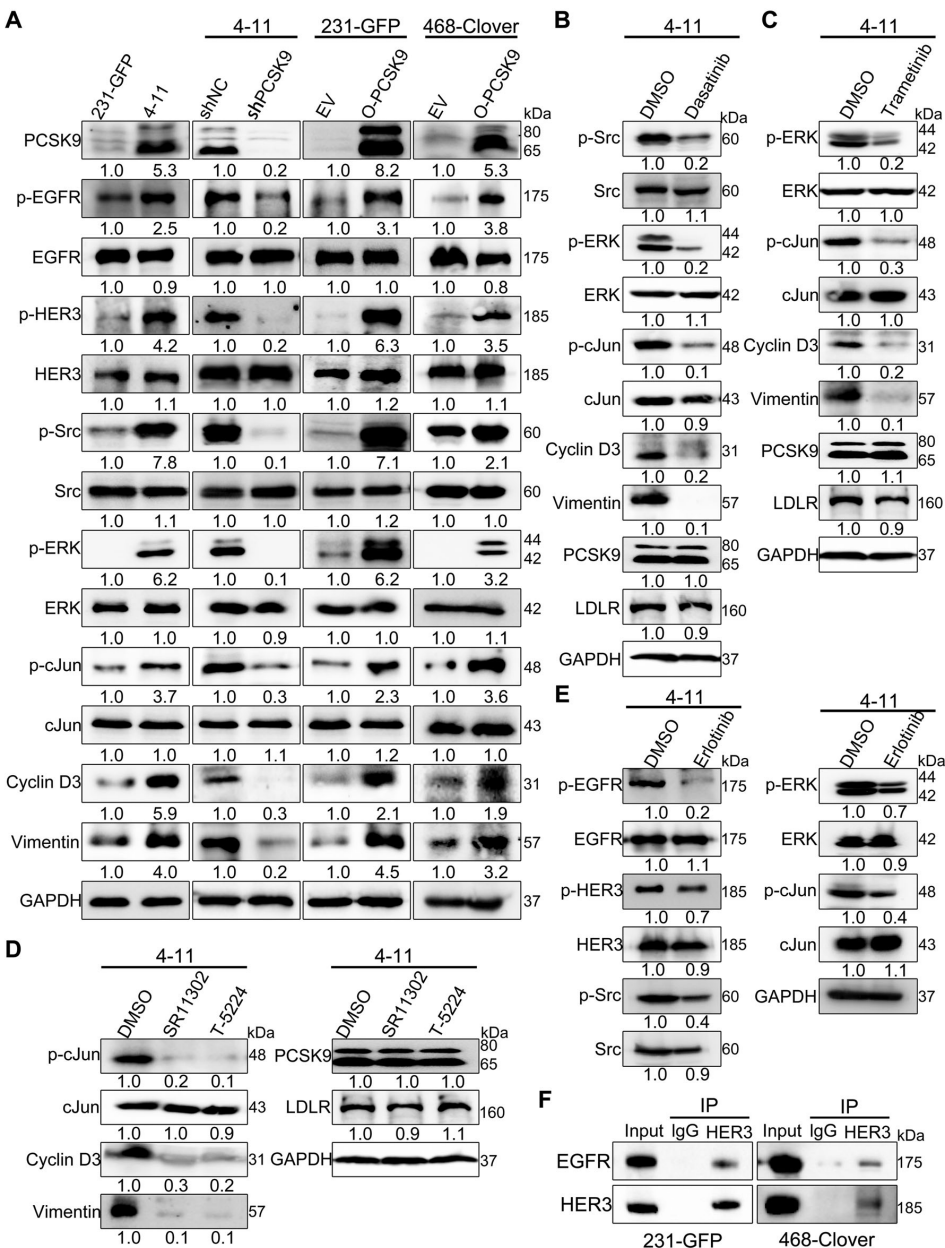
PCSK9 activated the EGFR/HER3 signaling pathway in TNBC cells. A) Western blotting depicting the phosphorylated or total protein levels of PCSK9, EGFR, HER3, Src, ERK, c‐Jun, cyclin D3, and vimentin in 231‐GFP and 4–11 cells, 4–11 cells of shNC or shPCSK9, and 231‐GFP and 468‐Clover cells with EV or O‐PCSK9. B) Western blotting results of p‐Src, Src, p‐ERK, ERK, p‐c‐Jun, c‐Jun, cyclin D3, vimentin, PCSK9 and LDLR in 4–11 cells after treatment with 50 nM dasatinib (Src inhibitor) for 48 h. C) Western blotting results of p‐ERK, ERK, p‐c‐Jun, c‐Jun, cyclin D3, vimentin, PCSK9 and LDLR in 4–11 cells after treatment with the MEK inhibitor trametinib (10 nM) for 48 h. D) Western blotting results of p‐c‐Jun, c‐Jun, cyclin D3, vimentin, PCSK9 and LDLR in 4–11 cells after treatment with the c‐Jun inhibitors SR11302 (5 µM) and T‐5224 (20 µM) for 48 h. E) Western blotting results of p‐EGFR, EGFR, p‐HER3, HER3, p‐Src, Src, ERK, p‐ERK, p‐c‐Jun, and c‐Jun in 4–11 cells after treatment with 10 µM erlotinib (EGFR inhibitor) for 48 h. F) Immunoprecipitation (IP) analysis of EGFR with the HER3 antibody.

Gene Ontology (GO) analysis revealed enrichment of cancer‐related pathways, such as those related to signal transduction, cell adhesion, cell population proliferation and the cell surface receptor signaling pathway, in 4–11 cells compared with 231‐GFP cells (Figure S5A, Supporting Information). To elucidate the mechanisms through which PCSK9 promotes cell proliferation and cancer metastasis, we examined the levels of multiple oncogenic proteins by Western blot analysis. Our results revealed clear increases in the phosphorylation of several key proteins, including phosphorylated epidermal growth factor receptor (p‐EGFR, 2.5‐fold), phosphorylated human epidermal growth factor receptor 3 (p‐HER3, 4.2‐fold), phosphorylated proto‐oncogene tyrosine‐protein kinase Src (p‐Src, 7.8‐fold), phosphorylated extracellular signal‐regulated protein kinases (p‐ERK, 6.2‐fold), and phosphorylated c‐Jun (p‐c‐Jun) (3.7‐fold), in 4–11 cells compared to 231‐GFP cells (Figure 5A). Additionally, we observed upregulation of cell cycle and epithelial–mesenchymal transition (EMT) markers, including cyclin D3 (CCND3, 5.9‐fold) and vimentin (VIM, 4.0‐fold), in 4–11 cells compared to 231‐GFP cells.

Although there was a slight increase in the mRNA levels of HER3 and EGF (epidermal growth factor) in 4–11 cells compared to 231‐GFP cells, there were no significant changes in the mRNA levels of EGFR, HER2 (human epidermal growth factor receptor 2), TGFA (transforming growth factor alpha), NRG1 (neuregulin 1), and NRG2 (neuregulin 2) in either 231‐GFP or 4–11 cells (Figure S5B, Supporting Information). No differences were detected in the mRNA levels of EGFR, HER2, HER3, EGF, TGFA, NRG1, or NRG2 (Figure S5C, Supporting Information). The qPCR results revealed that neither overexpression nor knockdown of PCSK9 affected the mRNA levels of EGFR, HER2, HER3, EGF, and TGFA in either 231‐GFP or 4–11 cells (Figure S5D, Supporting Information). In addition, the phosphorylation of other downstream signaling molecules of EGFR and HER3, such as p‐JNK, p‐p38, p‐PI3K, and p‐AKT, did not differ substantially between 231‐GFP and 4–11 cells (Figure S5E, Supporting Information). Overall, neither the overexpression nor the knockdown of PCSK9 significantly affected the protein levels of EGFR, HER2, and HER3. Moreover, alternations in PCSK9 expression did not change the mRNA levels of the ligands of EGFR (EGF, TGFA) or the ligands of HER3 (NRG1, NRG2).

To further elucidate the possible signaling molecules involved in PCSK9, we performed knockdown and overexpression experiments in 4–11, 231‐GFP and 468‐Clover cells. Then, we detected the protein levels of the involved genes via Western blotting. Upon knocking down PCSK9 in 4–11 cells, p‐EGFR, p‐HER3, p‐Src, p‐ERK, p‐c‐Jun, cyclin D3 and vimentin were significantly downregulated. We also confirmed that overexpressing PCSK9 activated these signaling molecules in the TNBC cell lines (231‐GFP and 468‐Clover) (Figure **5**A). We observed that the protein levels of p‐EGFR, p‐HER3, p‐Src, p‐ERK, p‐c‐Jun, cyclin D3 and vimentin increased 1.9‐ to 7.1‐fold than the control EV cells (Figure 5A).

Next, we treated 4–11 cells with dasatinib, a Src inhibitor, and observed a decrease in p‐Src levels, subsequently leading to reduced levels of p‐ERK, p‐c‐Jun, cyclin D3 and vimentin (Figure 5B). Subsequent treatment with three inhibitors, the mitogen‐activated protein kinase (MEK) inhibitor trametinib and the c‐Jun inhibitors SR11302 and T‐5224, effectively suppressed the activation of ERK and c‐Jun in 4–11 cells. MEK and c‐Jun inhibitors significantly inhibited p‐ERK, p‐cJun, cyclin D3 and vimentin (Figure 5C,D). Moreover, treating 4–11 cells with the Src inhibitor dasatinib, the ERK inhibitor trametinib, and the cJun inhibitors SR11302 and T‐5224 did not affect the protein levels of PCSK9 or LDLR, indicating these three kinases may function downstream of PCSK9 and LDLR (Figure 5B–D).

Although treating 4–11 cells with the EGFR inhibitor erlotinib greatly inhibited the phosphorylation of EGFR, it only partially reduced the phosphorylation of HER3 and its downstream molecules, including Src, ERK, and cJun (Figure 5E). In addition, the results of co‐Immunoprecipitation (Co‐IP) experiments showed that HER3 interacted with EGFR in 231‐GFP and 468‐Clover cells (Figure 5F). These results obtained thus far suggest that the high expression of PCSK9 in 4–11 cells may activate EGFR and HER3 in a homodimer and heterodimer fashion, thus inhibiting EGFR alone could not prevent its activation of the downstream Src‐ERK‐cJun‐cyclin D3‐vimentin pathway.

### 2.6 Targeting the EGFR/HER3‐Src‐ERK‐cJun Pathway Affected PCSK9‐Promoted Proliferative and Metastatic Phenotypes in TNBC Cells

These results in Figure 5 revealed that PCSK9 could activate the EGFR/HER3‐Src‐ERK‐cJun‐cyclin D3/vimentin signaling pathway in TNBC cells. We validated these findings by using specific inhibitors and activator via various in vitro and in vivo assays.

**Figure 6:**
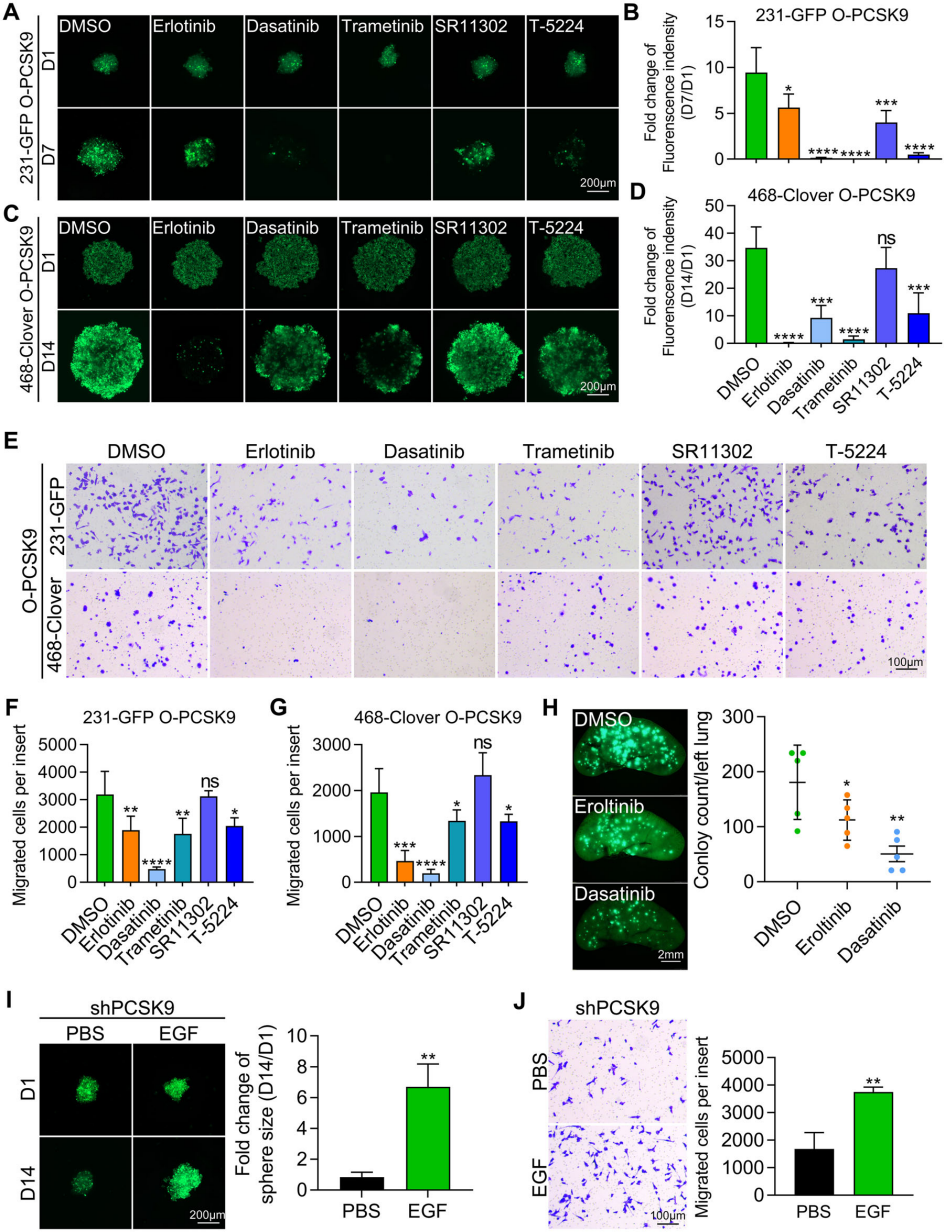
Inhibitors of EGFR, MEK, Src and cJun, and activator of EGFR/HER3 affected PCSK9‐promoted proliferation and metastasis in 231‐GFP and 468‐Clover cells. A,B) Representative images and quantified results of sphere formation from PCSK9‐overexpressing 231‐GFP cells treated with the inhibitors. Two thousand cells were seeded for 7 days. C,D) Representative images and quantified results of sphere formation from PCSK9‐overexpressing 468‐Clover cells treated with the inhibitors. Ten thousand cells were seeded for 14 days. E,F,G) Representative images and quantified results of transwell migration assay with ten thousand cells for 16 h. H) Representative images and quantified results of left lungs (*n* = 5). I) Representative images and quantified results of tumor spheres treated with EGF. Two thousand cells were seeded for 14 days. J) Representative images and quantified results of transwell migration assay treated with EGF. Ten thousand cells were cultured for 16 h. The significance of differences was determined by one‐way ANOVA (B, D, F, G, H) or Student’s *t‐test* (I, J).

We first used PCSK9‐overexpressing cells to test the effects of the inhibitors on sphere formation. PCSK9‐overexpressing 231‐GFP and 468‐Clover cells were treated with the EGFR inhibitor erlotinib (10 µM), the Src inhibitor dasatinib (50 nM), the MEK inhibitor trametinib (10 nM), and the c‐Jun inhibitors SR11302 (5 µM) and T‐5224 (20 µM). Fluorescence imaging and quantification revealed that most of the inhibitors, except SR11302, significantly reduced the sphere size by at least 40.3% (Figure **6**A–D). Additionally, a colony formation assay revealed that most inhibitors, except SR11302, significantly decreased colony formation ability (Figure S6A–‐C, Supporting Information).

Moreover, most of the inhibitors, except SR11302, significantly reduced the migration ability of the PCSK9‐overexpressing 231‐GFP and 468‐Clover cells (Figure 6E–G). Because EGFR and Src are the immediate downstream targets of PCSK9, we tested whether blocking EGFR or Src could affect PCSK9‐promoted lung colony formation in vivo. PCSK9‐overexpressing 231‐GFP cells were treated with the EGFR inhibitor erlotinib (10 µM) or the Src inhibitor dasatinib (50 nM) for 24 h. Half a million cells were injected into the tail vein of NOD/SCID mice, and photos of the left lungs were taken after 28 days to determine the number of colonies. Erlotinib and dasatinib treatment exhibited 60.4% and 96.0% reductions in the number of lung colonies, respectively (Figure 6H).

We also used EGF, a well‐known growth factor of EGFR, to activate EGFR in PCSK9 knockdown cells. We first used Western blotting to show that treating shPCSK9 cells with 25 nM EGF for 4 h could increase the phosphorylation levels of EGFR and HER3 for 2.4‐fold and 1.9‐fold, respectively (Figure S6D, Supporting Information). These results suggested that EGF can activate both EGFR and HER3, which has also been reported in a paper published in *PNAS* (2017).^[^ ^34^ ^]^

Next, we treated shPCSK9 cells with 25 nM EGF and found that EGF significantly increased cell growth and migration abilities (Figure 6I,J, and Figure S6E, Supporting Information). Specifically, EGF treatment reversed the knockdown effect of PCSK9 in 4–11 cells by increasing the sphere size 8.0‐fold (Figure 6I), the number of colonies 2.2‐fold (Figure S6E, Supporting Information), and the number of migrated cells 2.2‐fold (Figure 6J). In conclusion, targeting the EGFR/HER3‐Src‐ERK‐cJun pathway with specific inhibitors and activator of EGFR/HER3 could significantly affect PCSK9‐promoted proliferative and metastatic abilities in TNBC cells.

### 2.7 PCSK9 Promoted the Degradation of Cell‐Surface LDLR and Reduced Cholesterol Levels at the Cell Membrane

**Figure 7:**
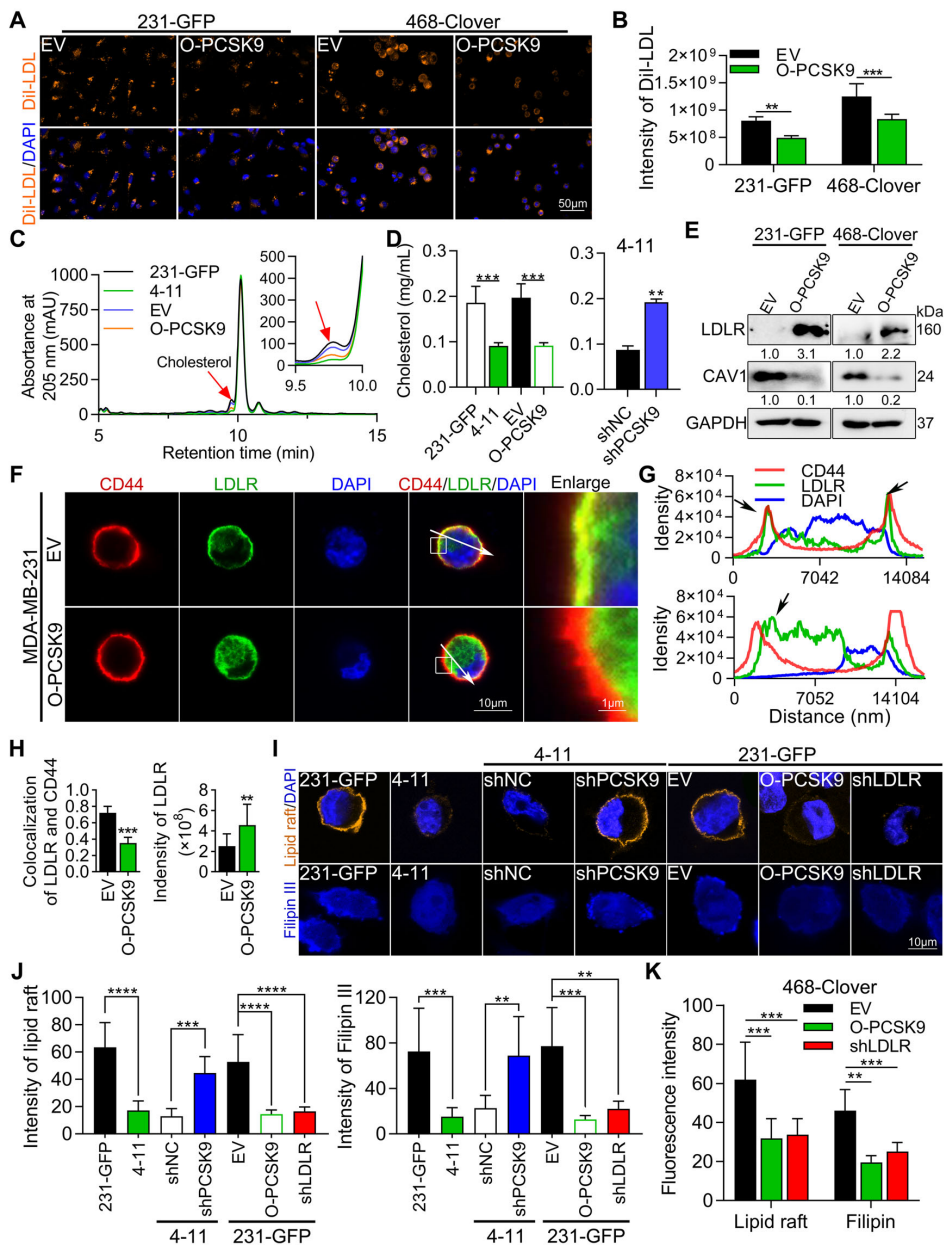
High expression of PCSK9 reduced cholesterol levels in the cell membrane by decreasing the cell‐surface LDLR. A,B) Representative images (A) and quantified results (B, n ≥ 100 cells) of DiI‐LDL in 231‐GFP and 468‐Clover cells with EV or O‐PCSK9. C) The contents of cholesterol in 231‐GFP, 4–11 and O‐PCSK9 cells using a sensitive high performance liquid chromatography (HPLC)‐based method. D) Quantified results of cholesterol levels by HPLC. E) Western blotting showed the protein levels of LDLR and CAV1 after overexpressing PCSK9 in 231‐GFP and 468‐Clover cells, respectively. F) Coimmunostaining of LDLR and CD44 in MDA‐MB‐231 cells with EV and O‐PCSK9. The white arrow indicates the coordinates (G) used for the intensity profiles of scan line. G) The coordinates for intensity profiles of scan line were shown for colocalization of CD44, LDLR and DAPI in the EV (top) and O‐PCSK9 (bottom) groups of MDA‐MB‐231 cells. (H, n ≥ 50 cells) Ratio of colocalization LDLR and CD44 (left) and fluorescence intensity of LDLR (right). I,J) Representative images (I) and quantified results (J, n ≥ 20 cells) of lipid rafts and filipin III staining in 231‐GFP cells. (K, n ≥ 20 cells) Quantified results of lipid raft and Filipin III staining in 468‐Clover cells. The significance of differences was determined by one‐way ANOVA (D, J), two‐way ANOVA (B, K) or Student’s *t‐test* (D, H).

Cholesterol can bind with LDL to form an LDL cholesterol complex, which can bind to LDLR at the plasma membrane for transport into cells. The main function of PCSK9 is to bind with LDLR in the plasma membrane to trigger its internalization. We thus hypothesized that high expression of PCSK9 might reduce the level of LDLR at the plasma membrane, resulting in a decreased a uptake of cholesterol.^[^ ^35^, ^36^ ^]^ To confirm these findings, we used DiI‐low density lipoprotein (human DiI‐LDL) to treat 231‐GFP and 468‐Clover cells with EV or O‐PCSK9 for 4 h (Figure **7**A). The results revealed that overexpression of PCSK9 decreased the cytoplasmic DiI‐LDL level by ≈38.6% and 33.3%, respectively, in 231‐GFP and 468‐Clover cells (Figure 7B). These results confirmed that the overexpression of PCSK9 reduced LDL uptake in TNBC cells. To assess cellular cholesterol levels in 231‐GFP, 4–11, and O‐PCSK9 cells, we employed a high‐performance liquid chromatography (HPLC)‐based method after extracting lipids using methanol/chloroform.^[^ ^37^ ^]^ The HPLC assay demonstrated linear detection across a wide range of concentrations (0.01 to 2 mg mL^−1^) with excellent precision (Figure S7A, Supporting Information). Our HPLC measurements revealed a significant reduction in cholesterol levels in 4–11 and O‐PCSK9 cells (Figure 7C), with cholesterol content 51.2% and 53.9% lower, respectively, compared to 231‐GFP cells (Figure 7D, left). Consistently, cholesterol levels in shPCSK9 cells were 54.5% higher than in 4–11 negative control cells (Figure 7D, right). Additionally, those dried lipids were measured by cholesterol test kit and the level of cholesterol in 231‐GFP cells was ≈60% more than the levels of O‐PCSK9 cells (Figure 7C). These results suggest that high expression of PCSK9 may be correlated with decreased cholesterol levels of the cells.

Next, we investigated whether the high expression of PCSK9 could affect the expression of LDLR. The overexpression of PCSK9 resulted in increased LDLR protein levels in the cytoplasm of both 231‐GFP and 468‐Clover cells (Figure 7E). Cluster of differentiation 44 (CD44) is a highly abundant cell‐surface glycoprotein.^[^ ^38^ ^]^ Coimmunostaining of CD44 and LDLR was used to observe the plasma localization of these two proteins. Fluorescence imaging and quantified results showed that although overexpression of PCSK9 increased expression of LDLR within the MDA‐MB‐231 cells by 1.8‐fold, it resulted in a significant 51% reduction in LDLR levels on the plasma membrane (Figure 7F–H). We further quantified the fluorescence intensity across the cells along the scan line indicated by the white arrow line in Figure 7F. The results of line profile showed that in EV‐MDA‐MB‐231 cells, the signals of LDLR and CD44 were both peaked on the cell surface, as indicated by the two black arrows in the top panel of Figure 7G. In contrast, in PCSK9‐overexpressing MDA‐MB‐231 cells, the CD44 signal still peaked on both sides of the cell surface just like the profile in the control cells, while the LDLR signal shifted from the cell surface to inside the cell (Figure 7G, bottom panel).

We then used qPCR to find out whether overexpressing PCSK9 could increase the LDLR mRNA expression in 231‐GFP cells. The qPCR results showed that overexpression of PCSK9 slightly increased mRNA levels of LDLR, and sterol regulatory element‐binding protein 1 (SREBP1) which controls the transcription of both LDLR and PCSK9 (Figure S7C, left, Supporting Information). We designed the primers of *srebp1a* and *srebp1c* though NCBI primer design tool and observed that overexpressing the expression of PCSK9 in 231‐GFP or 468‐Clover cells did not affect the expression of these two genes (Figure S7C, middle and right, Supporting Information).We further validated that reducing the expression of PCSK9 in 4–11 cells, also did not affect mRNA levels of SREBP1 and SREBP2 (Figure S7D, Supporting Information).

So far, we have found that overexpression of PCSK9 reduces the protein level of LDLR in the plasma membrane, leading to decreases in LDL uptake and alterations of cholesterol levels in plasma membrane. Given that lipid rafts in the plasma membrane are enriched in cholesterol and that 40% to 60% of EGFR is located in lipid rafts, we evaluated the lipid raft‐related protein caveolin‐1 (CAV1) through immunostaining and Western blotting analysis.^[^ ^39^, ^40^, ^41^ ^]^ The fluorescence images obtained after coimmunostaining of LDLR and CAV1, revealed a significant decrease of ≈67% in CAV1 expression in MDA‐MB‐231 cells overexpressing PCSK9 (Figure S7F, Supporting Information). Interestingly, overexpressing PCSK9 greatly reduced the protein level of CAV1 in both 231‐GFP and 468‐Clover cells (Figure 7E) However, these reduction may not resulted from reducing the mRNA levels of CAV1 as changing the expression of PCSK9 by overexpression or silencing did not affect the mRNA level of CAV1 in either 231‐GFP or 4–11 cells (Figure S7E, Supporting Information). Clinical database analysis of the cBioPortal and UALCAN platforms further revealed that breast cancer and TNBC patients have lower levels of CAV1 protein (Figure S7G,H Supporting Information). In summary, our data showed that PCSK9 could decrease the lipid raft‐related protein CAV1 and that lower level of CAV1 protein is associated with TNBC.

Next, we investigated the effects of PCSK9 and LDLR on lipid rafts and cholesterol levels using lipid raft kit and Filipin III staining. Initially, significant decreases of ≈73% and 79% in lipid rafts and cholesterol levels, respectively, were detected in 4–11 cells compared to 231‐GFP cells (Figure 7I,J). After knocking down PCSK9 in 4–11 cells, we observed dramatic increases in lipid raft and cholesterol levels, which were ≈3.4‐fold and 3.0‐fold higher compared to shNC, respectively. Furthermore, our study explored the effects of PCSK9‐overexpressing and LDLR‐knockdown on lipid rafts in 231‐GFP cells. Our findings indicated that both resulted in significant decreases of lipid raft and cholesterol levels in the 231‐GFP cells. Quantified results revealed decreases of lipid raft and cholesterol levels in both overexpression of PCSK9 and knockdown of LDLR cells (Figure 7J). Notably, similar results were observed after overexpression of PCSK9 and knockdown of LDLR in 468‐Clover cells (Figure 7K). These findings highlight that PCSK9 promotes the degradation of cell‐surface LDLR and reduces lipid rafts and cholesterol levels in the cell membrane, potentially influencing TNBC pathogenesis.

### 2.8 Decreased Lipid Rafts in the Plasma Membrane Led to the Activation of EGFR and HER3 to Promote Proliferation and Metastasis

We next investigated the effects of decreased lipid rafts in the plasma membrane on EGFR/HER3 activation and its influence on the proliferation and metastasis of TNBC cells. We used methyl‐β‐cyclodextrin (MβCD) to deplete lipid rafts and performed colony formation and transwell migration assays in vitro.

**Figure 8:**
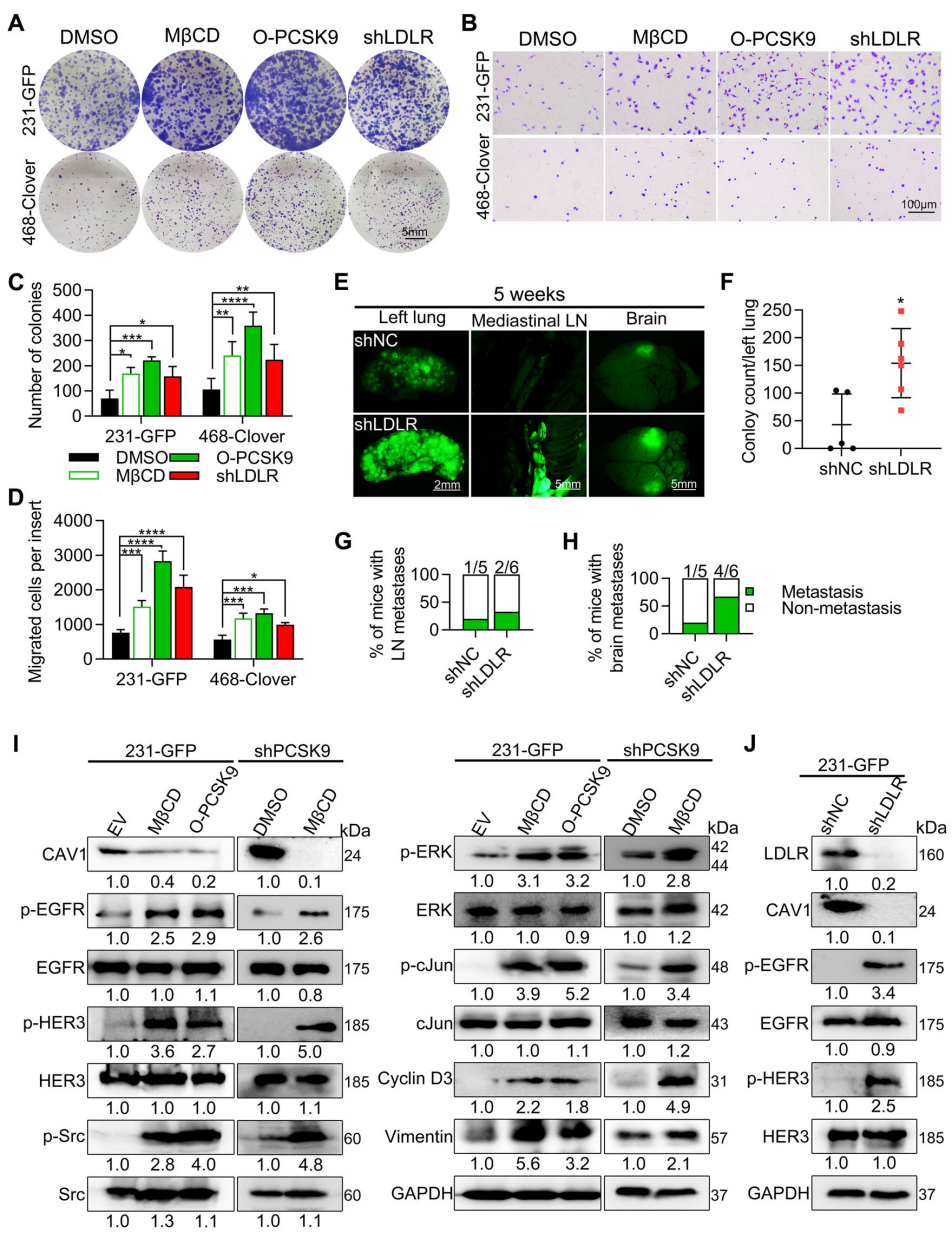
PCSK9 activated EGFR and HER3 by reducing lipid rafts on cell membranes. A) Representative images and quantified results of colony formation for the EV, MβCD‐treated, PCSK9‐overexpressing and LDLR‐knockdown groups. One thousand 231‐GFP cells were cultured in 6‐well plates for 12 days, and five thousand 468‐Clover cells were cultured in 6‐well plates for 14 days. B) Representative images and quantified results of the transwell migration assay for the EV, MβCD‐treated, PCSK9‐overexpressing and LDLR‐knockdown groups in 231‐GFP cells and 468‐Clover cells. Five thousand cells were cultured for 16 h in migration assays. C,D) Quantified results of the colony formation and transwell migration assays. E) Representative images of left lung colonies and metastatic tumors after 5 weeks (*n* = 5–6). F) Quantified results of left lung colonies. G) Quantified results of metastatic tumors in mediastinal LN. H) Quantified results of metastatic tumors in brain. I) Western blotting depicting the phosphorylated and total protein levels of CAV1, EGFR, HER3, Src, ERK, c‐Jun, cyclin D3, and vimentin in the EV, MβCD‐treated and O‐PCSK9 groups of 231‐GFP cells, and in the DMSO‐treated and MβCD‐treated groups of 4–11 cells with shPCSK9. J) Western blot showed the phosphorylated and total protein levels of EGFR, HER3, LDLR and CAV1 after LDLR was knocked down in 231‐GFP cells. The significance of differences was determined by two‐way ANOVA (C, D) or Student’s *t‐test* (F).

We first assessed the efficacy of MβCD treatment in reducing the cholesterol content of cell membranes. Compared with those in the control group, the cholesterol levels in the 2 mM MβCD treatment group or the PCSK9 overexpression group were effectively reduced in the 231‐GFP cells (Figure S7B, Supporting Information). In the colony formation assay, the number of colonies increased 2.1‐fold and 3.1‐fold with MβCD treatment and PCSK9 overexpression, respectively, in the 231‐GFP cells (Figure **8**A,C). Similarly, in 468‐Clover cells, the number of colonies increased 2.3‐fold and 3.4‐fold, respectively. When LDLR was knocked down in 231‐GFP cells, the number of colonies increased 1.9‐fold. In the 468‐Clover cells, the number of colonies also increased 1.9‐fold (Figure 8A,C). These results indicated that lipid raft depletion significantly enhanced colony formation. Moreover, notable increases in the number of migrated cells were observed compared to that in the 231‐GFP group, with MβCD treatment, PCSK9 overexpression and LDLR knockdown in 231‐GFP and 468‐Clover cells (Figure 8B). The quantified results revealed significant increases in cell migration upon cholesterol depletion, PCSK9 overexpression and LDLR knockdown (Figure 8D).

To further investigate the role of LDLR in TNBC metastasis, half a million shLDLR cells were injected into the tail vein of nude mice. After 5 weeks, the mice were euthanized, and their organs, bones, and tissues were examined for signs of metastasis. Fluorescence imaging revealed metastases in the mediastinal lymph nodes (LN) and brain in the shLDLR group (Figure 8E). Additionally, the number of colonies formed in the left lung was over 3‐fold higher in the shLDLR group compared to the controls (Figure 8F). Quantification of metastatic tumors formed in each mice group showed that 33.3% of the shLDLR group developed metastases in the mediastinal LN, while 66.7% of the mice exhibited brain metastases (Figure 8G,H). These results highlight the significant role of LDLR in enhancing the proliferative and metastatic potentials of TNBC cells.

Furthermore, in 231‐GFP cells, treatment with MβCD and overexpression of PCSK9 led to significant activation of five kinases and downstream molecules, including EGFR, HER3, Src, ERK, c‐Jun, cyclin D3 and vimentin as well as decreased protein levels of CAV1 (Figure 8I). In 4–11 cells with PCSK9 knockdown, treatment with MβCD restored the activation of the same signaling pathway, highlighting the role of PCSK9 and lipid rafts in activating these signaling molecules (Figure 8I). Moreover, we observed decreased protein levels of CAV1 in 4–11 cells following MβCD treatment, which is consistent with our previous findings that lipid raft depletion activates the EGFR/HER3 signaling pathway. The knockdown of LDLR in 231‐GFP cells resulted in reduction of CAV1 and activation of p‐EGFR and p‐HER3, further indicating the involvement of LDLR in lipid raft‐mediated activation of these receptors (Figure 8J).

To further investigate the effect of CAV1, we knocked down and overexpressed CAV1 in TNBC cells, respectively. The knockdown of CAV1 significantly increased the migration ability of 231‐GFP cells (Figure S8A–B, Supporting Information). Consistently, overexpression of CAV1 inhibited colony formation in both 231‐GFP and 468‐Clover cells under the condition of overexpressing PCSK9 (O‐PCSK9) (Figure S8C,D, Supporting Information). However, in 231‐GFP cells, overexpression of CAV1 only reduced the migration ability of 231‐GFP cells but not 468‐Clover cells (Figure S8C,D, Supporting Information), which is in agreement with the shCAV1 effects shown in Figure S8A,B, Supporting Information. In 4–11 cells with shPCSK9, CAV1 knockdown also greatly increased cell migration (Figure S8E,F, Supporting Information). These results suggest that CAV1 acts as a suppressor of proliferation and migration in TNBC cells.

**Figure 9:**
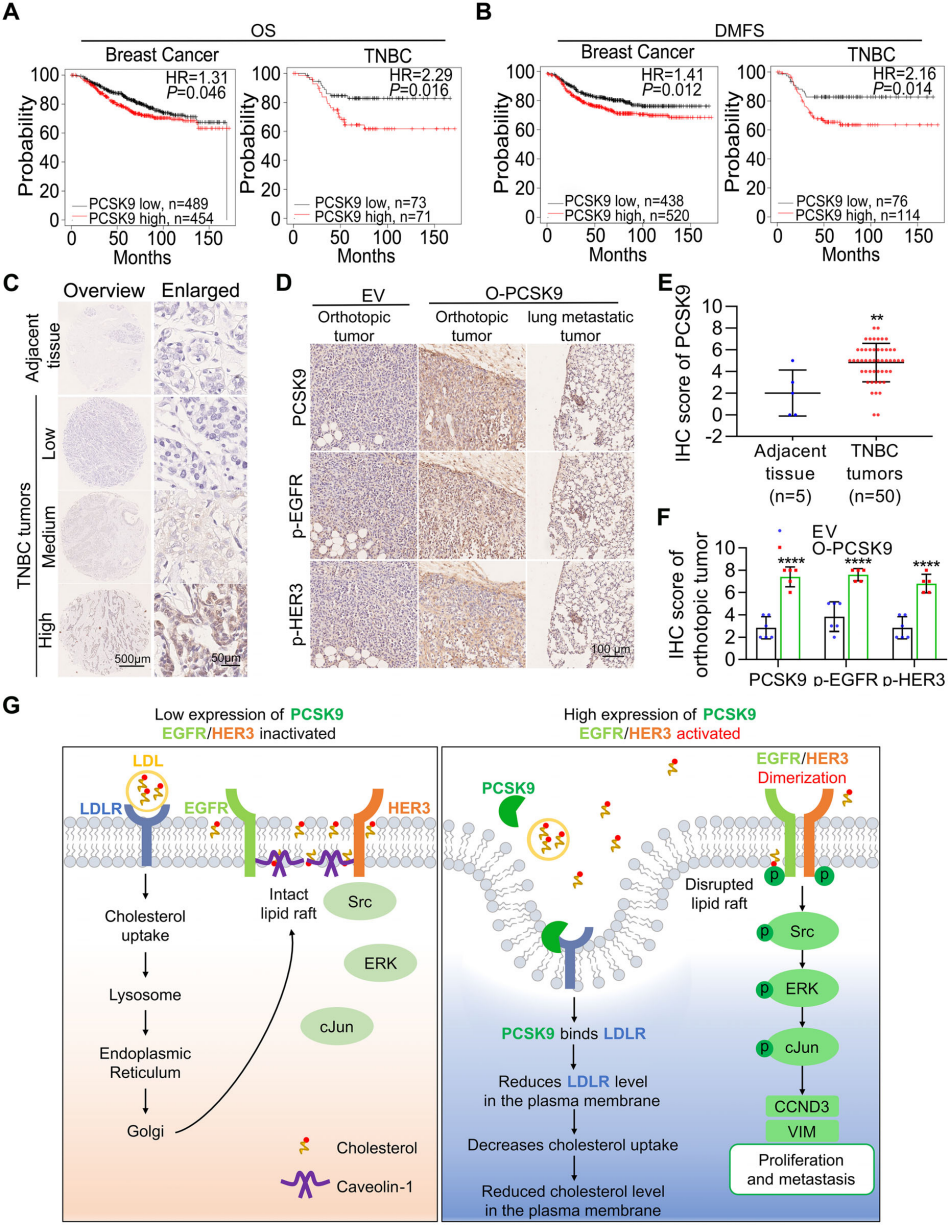
High expression of PCSK9 is associated with a poor clinical prognosis, and the EGFR/HER3 pathway is activated by high expression of PCSK9 to promote the proliferation and metastasis of TNBC cells. A) Kaplan–Meier plots of OS curves in breast cancer patients and TNBC patients stratified by PCSK9 expression. B) DMFS curves in breast cancer patients and TNBC patients grouped based on PCSK9 expression. C) Representative overview or enlarged images of IHC staining obtained from samples of TNBC patients categorized based on PCSK9 expression. D) Representative images of IHC staining of serial sections categorized based on PCSK9, p‐EGFR and p‐HER3 expression. E) Quantified IHC scores of PCSK9 were determined in adjacent tissues and TNBC tumor tissues. F) Quantified IHC scores of PCSK9, p‐EGFR and p‐HER3 were determined in orthotopic tumors of EV and O‐PCSK9. G) Under normal conditions, LDLR facilitates the uptake of LDL from the bloodstream, contributing to lipid raft formation in the cell membrane (left). In the presence of high‐expressing PCSK9, the levels of LDLR are reduced at the cell surface, resulting in decreased cholesterol levels in the plasma membrane and disruption of lipid raft. This disruption facilitates the dimerization of EGFR and HER3 which activates the Src/ERK/c‐Jun signaling cascade, resulting in the upregulation of cyclin D3 and vimentin to promote cell proliferation and metastasis in TNBC cells (right). The significance of differences was determined by two‐way ANOVA (F) and Student’s *t‐test* (E).

Moreover, reducing the expression of CAV1 resulted in a reduction of LDLR level at the cell membrane and an increase in LDLR level in the cytoplasm (Figure S8H, Supporting Information). This redistribution of LDLR suggests that CAV1 may influence the trafficking and localization of lipid raft components, including LDLR. Previous studies have shown that depletion of CAV1 in mice reduces LDL uptake.^[^ ^42^, ^43^ ^]^ A lower protein level of CAV1 has been associated with the activation of receptor tyrosine kinase (RTK) pathways in breast cancer patients (*n* = 56), suggesting potential clinical relevance for these molecular events (Figure S8G, Supporting Information). Taken together, the overexpression of PCSK9 reduced lipid raft content and activated the EGFR/HER3 pathway, contributing to increased proliferation and metastasis in TNBC cells (**Figure** **9**G).

### 2.9 High Expression of PCSK9 is Correlated with a Poor Prognosis in TNBC Patients

To explore the potential clinical relevance of PCSK9 expression and disease progression in TNBC patients, we first used Kaplan–Meier analysis. The graphs revealed that high levels of PCSK9 were linked to shorter overall survival (OS) and distant metastasis‐free survival (DMFS) in all breast cancer patients and TNBC patients. Notably, TNBC patients exhibited lower survival rates than did all breast cancer patients (Figure 9A,B). Although high HER3 expression was linked to a better prognosis in all breast cancer patients, it was significantly correlated with lower OS and DMFS rates in TNBC patients (Figure S9A,B, Supporting Information). Additionally, low levels of CAV1 were correlated with shorter DMFS in TNBC patients (Figure S9C,D, Supporting Information). Moreover, metadata analysis also showed a positive correlation between p‐HER3 proteins and PCSK9 mRNA in samples of TNBC patients from the cBioPortal platform (Figure S9E, Supporting Information).

Next, we performed an immunohistochemistry (IHC) staining assay on samples from TNBC patients, including adjacent tissues (*n* = 5) and TNBC tumors (*n* = 50) (Figure 9C). The results revealed that the IHC scores of PCSK9 in tumor tissues were significantly higher than those in the adjacent tissues, increasing by 2.48‐fold (Figure 9E). We also analyzed primary tumors and lung metastatic tumors derived from PCSK9‐overexpressing 231‐GFP cells that were implanted in the mammary fat pads of NOD/SCID mice. IHC staining of serial sections of the orthotopic tumors and lung metastatic samples (*n* = 5) displayed significantly higher levels of PCSK9 and the activation of EGFR and HER3 (Figure 9D,F).

## 3 Discussion

In this study, we conducted a comprehensive investigation regarding how PCSK9 can promote tumorigenesis and metastasis of TNBC. Our findings revealed that the high expression of PCSK9 reduced LDLR at the plasma membrane, which decreased LDL cholesterol uptake and resulted in a significant reduction in lipid rafts together with caveolin‐1 protein at the plasma membrane. Subsequently, the reduced lipid rafts facilitated the activation of EGFR and HER3 via homo or heterodimerization which further activated their downstream signaling molecules including Src, ERK and c‐Jun to increase the expression of cyclin D3 and vimentin increasing the proliferation and metastasis of TNBC cells (Figure 9G).

PCSK9 belongs to a family of proprotein convertases with 9 family members including PCSK1‐2, Furin, and PCSK4‐9. These convertases can cleave precursor proteins into their active forms and play critical roles in regulating various physiological processes, including cell proliferation, cell death, and immune responses.^[^ ^44^, ^45^ ^]^ PCSK9, can bind to the epidermal growth factor repeat A (EGF‐A) on the cell surface LDLR, resulting in its internalization and degradation in lysosomes. As LDLR can bind to LDL to take up cholesterol from outside cells, PCSK9 can therefore negatively regulate the levels LDLR as well as cholesterol at the plasma membrane. It has been reported that PCSK9 inhibited LDLR‐mediated cholesterol uptake by live cells, which resulted in an increased level of LDL cholesterol in the blood.^[^ ^46^ ^]^ Gain‐of‐function mutations in PCSK9 increased LDL cholesterol levels in the blood and elevated cardiovascular risk, while loss‐of‐function mutations decreased plasma levels of LDL cholesterol and reduced cardiovascular risk.^[^ ^47^, ^48^ ^]^ Furthermore, studies have reported that the levels of PCSK9 were elevated in the blood samples of stage III breast cancer patients compared to healthy controls.^[^ ^49^ ^]^ In the future study, it would be valuable to explore plasma PCSK9 levels specifically in TNBC patients compared to other breast cancer subtypes and healthy individuals to elucidate any potential differences and their clinical significance.

In this study, we established three metastatic model cell lines which were derived from lung colonies in nude mice after parental 231‐GFP cells were introduced into the nude mice via tail vein injection. Among the three metastatic cell lines from one mouse, 4–11 showed the highest growth rate compared to the 231‐GFP parental cells. Subsequent RNA‐seq analysis revealed a significant upregulation of PCSK9, highlighting its importance in facilitating the tumorigenic and metastatic phenotypes observed in TNBC cells. Our findings in TNBC align with some of the prior studies indicating that increased PCSK9 expression is associated with increased tumor growth and metastasis in other types of cancers. Specifically, studies have demonstrated that PCSK9 could interact with major histocompatibility complex I (MHCI) proteins, leading to their degradation and subsequently contributing to immune escape in B16F10, CT26, 4T1, MC38 and MDA‐MB‐231 cells.^[^ ^29^ ^]^ Investigations on hepatocellular carcinoma and gastric cancer have revealed that PCSK9 overexpression promoted tumor growth by inhibiting apoptosis, correlating with poor clinical outcomes.^[^ ^28^, ^50^ ^]^ Similarly, in colon cancer, PCSK9 promoted proliferation and metastasis through PI3K/AKT pathway activation and EMT induction, while also influenced M2 macrophage polarization to modulate antitumor immunity.^[^ ^51^ ^]^ Furthermore, PCSK9 bound to LDLR, impairing interaction of LDLR and T‐cell receptor (TCR) complex and TCR signaling. Then, PCSK9 inhibition might enhance CD8^+^ T‐cell antitumor activity, which is a promising cancer immunotherapy target.^[^ ^52^ ^]^ Additionally, PCSK9 activated the KRAS/MEK/ERK pathway by inducing cholesterol and geranylgeranyl pyrophosphate (GGPP) biosynthesis, through its binding with LDLR, leading to the dysregulation of cholesterol homeostasis, in APC/KRAS‐mutant colorectal cancer.^[^ ^53^ ^]^ Overall, augmenting PCSK9 expression has been shown to enhance both tumor growth and metastasis.

Although the function of PCSK9 in binding with LDLR to reduce LDL uptake has been recognized, its involvement in promoting tumor development primarily seems to revolve around the anti‐tumor immunity pathway or negative feedback to activate cholesterol synthesis. In our study, injection of TNBC cells with either knockdown or overexpression of PCSK9 into NOD/SCID mice resulted in significant decreases and increases in orthotopic and metastatic tumor sizes compared to those in the control group. Notably, NOD/SCID mice lack mature T and B cells and have defective NK cell functions. Therefore, we believe that the oncogenic function of PCSK9 may involve not only immune‐related pathways but also other significant mechanisms.

Our findings indicated that PCSK9 might enhance the activation of EGFR and HER3, even in the absence of changes in their expression levels. It was reported that the observed potent decrease in TNBC tumor growth upon treatment with a combination of a HER3 inhibitor and an EGFR inhibitor,^[^ ^54^ ^]^ which suggests that simultaneous activation of EGFR and HER3 may contribute to tumor growth, implicating a potential role for PCSK9 in modulating the activity or localization of these receptors on the cell surface. Therefore, further investigation into the basic biological functions of PCSK9 is warranted.

In our study, we assessed lipid raft, which is cholesterol‐rich areas in cell membranes. While cholesterol is essential for cellular processes, excessive cholesterol can be harmful. For example, excessive cholesterol can decrease membrane fluidity and lead to the generation of harmful oxidative molecules such as oxysterols.^[^ ^55^ ^]^ Thus, the intracellular cholesterol level must be carefully regulated to maintain a proper balance. Several studies have demonstrated that cholesterol depletion from the plasma membrane, often induced by MβCD treatment, triggered a signal transduction through a ligand‐independent activation of EGFR, leading to phosphorylation of downstream targets. This process involves a change in bilayer thickness that promotes a transition from less stable transmembrane (TM) domain dimers to the most stable N‐crossing TM dimer configuration. These results are also applicable to other HER family members.^[^ ^56^ ^]^ Additionally, MβCD treatment has been shown to increase the number of dimers in cells harboring resistant EGFR mutations (such as L858R and T790 M) against first‐ and second‐generation tyrosine kinase inhibitors (TKIs).^[^ ^57^ ^]^ The modulation of HER family downstream signaling and the stability and localization of HER2 on the cell membrane can be influenced by the temporal depletion of cholesterol, with caveolin‐1 (CAV1) playing a regulatory role.^[^ ^58^, ^59^ ^]^ Overall, these findings suggest a close association between the HER family members and lipid rafts, with their activation often requiring release from these specialized membrane microdomains. While the precise molecular mechanisms underlying these interactions remain unclear, it is hypothesized that the significant conformational changes necessary for EGFR and HER3 activation may be constrained within lipid rafts.

In this study, we found that cholesterol levels in the membranes decreased with the overexpression of PCSK9 and the knockdown of LDLR. To validate the impact of cholesterol clearance in the membranes, we employed a cholesterol inhibitor (MβCD). Confirming our hypothesis, we observed that the removal of cholesterol from the membranes promoted the proliferation and metastasis of TNBC cells in vitro. These findings underscore the intricate relationships among PCSK9, LDLR, and cellular cholesterol dynamics, providing valuable insights into potential pathways for therapeutic intervention in TNBC.

Despite the limited clinical utility of EGFR inhibitors in treating TNBC, our research provides insights into this conundrum by elucidating the role of PCSK9 in lipid raft signaling and EGFR/HER3 pathway. Targeted therapeutic strategies against HER3 may hold promises for treating triple‐negative breast cancer. As the high expression of PCSK9 can activate HER3/EGFR to promote tumor growth and metastasis, therefore reducing PCSK9 level may be a good strategy for treating TNBC. In the current study, we have knocked down PCSK9 using short hairpin RNA (shPCSK9) and verified that PCSK9 knockdown blocked EGFR/HER3 pathways, further reduced the proliferation and metastatic abilities of TNBC cells. Based on those findings, we proposed that PCSK9 inhibitors might be used alone or in combination with EGFR and HER3 inhibitors to treat patients with TNBC.

## 4 Experimental section

### Cell Lines and Culture

The breast cancer cell lines (MDA‐MB‐231, MDA‐MB‐468, UACC‐893, SK‐RB‐3, and MCF‐7) along with 293T cells were obtained from the American Type Culture Collection (ATCC, Manassas, VA). Additionally, the 4‐3, 4–5, and 4–11 cell lines were derived from lung metastatic tumors in female nude mice injected with MDA‐MB‐231‐GFP (231‐GFP) cells via the tail vein, as previously described.^[^ ^31^, ^60^ ^]^ All of the cell lines (MDA‐MB‐231, MDA‐MB‐468, UACC‐893, SK‐RB‐3, MCF‐7, 293T, 4‐3, 4–5 and 4–11) were cultured in Dulbecco's modified Eagle's medium (DMEM; #12 100 046, Thermo Fisher Scientific, USA), supplemented with 10% fetal bovine serum (FBS) (#10270‐106, Gibco, USA) and 1% penicillin‐streptomycin (PS) (#15 140 122, Thermo Fisher Scientific, USA). Culturing was performed at 37 °C in a modified incubator with 5% CO_2_. Specifically, 231‐GFP, 4‐3, 4–5, and 4–11 cells were transfected with the gene encoding green fluorescent protein (GFP) from Dr. Renfei Wu in our laboratory.^[^ ^31^ ^]^ 468‐Clover cells were transfected to induce constitutive expression of the green fluorescent protein Clover from Dr. Meng Hao of in our laboratory.^[^ ^61^ ^]^

### Reagent

All reagents, including the EGFR inhibitor erlotinib, Src inhibitor dasatinib, MEK inhibitor trametinib, AP‐1 inhibitors T‐5224 and SR‐11302 and methyl‐β‐cyclodextrin (MβCD), were dissolved in dimethyl sulfoxide (DMSO).

### Antibodies

The antibodies used in this study were as follows: anti‐PCSK9 (Cell Signaling Technology, Cat# 85813S, 1:1000, 1:100), anti‐PCSK9 (Abcam, Cat# ab28770, 1:100), anti‐LDLR (Abcam, Cat# ab30532, 1:1000), anti‐phospho‐EGFR (Cell Signaling Technology, Cat# 2220S, 1:1000), anti‐phospho‐EGFR (Cell Signaling Technology, Cat# 3777S, 1:100), anti‐EGFR (Cell Signaling Technology, Cat# 4267S, 1:1000), anti‐EGFR (Absin, Cat# abs149686, 1:1000), anti‐phospho‐HER3 (Cell Signaling Technology, Cat# 4561S, 1:1000), anti‐phospho‐HER3 (Cell Signaling Technology, Cat# 4791S, 1:100), anti‐HER3 (Cell Signaling Technology, Cat# 12708S, 1:1000), anti‐phospho‐Src (Cell Signaling Technology, Cat# 6943S, 1:1000), anti‐Src (Cell Signaling Technology, Cat# 6943S, 1:1000), anti‐phospho‐ERK (Cell Signaling Technology, Cat# 4695, 1:1000), anti‐ERK (Cell Signaling Technology, Cat# 4370S, 1:1000), anti‐phospho‐c‐Jun (Cell Signaling Technology, Cat# 3270S, 1:1000), anti‐c‐Jun (Cell Signaling Technology, Cat# 9165, 1:1000), anti‐cyclin D3 (Cell Signaling Technology, Cat# 2936, 1:1000), anti‐vimentin (Cell Signaling Technology, Cat# 5741S, 1:1000), anti‐CAV1 (Cell Signaling Technology, Cat# 3267, 1:1000), anti‐CAV1 (Abcam, Cat# ab17052, 1:100), anti‐CD44 (Cell Signaling Technology, Cat# 3570S, 1:100), anti‐IgG (Cell Signaling Technology, Cat#3900, 1:100) anti‐GAPDH (Cell Signaling Technology, Cat# 2118, 1:1000), goat anti‐rabbit IgG (H+L) Secondary Antibody Alexa Fluor Plus 594 (Invitrogen, Cat#A11037, 1:100), goat anti‐rabbit IgG (H+L)‐HRP secondary antibody (Bio‐Rad, Cat#1 706 515, 1:5000), goat anti‐mouse IgG (H+L)‐HRP secondary antibody (Bio‐Rad, Cat#1 706 516, 1:5000), rabbit anti‐goat IgG (H+L)‐HRP secondary antibody (Bio‐Rad, Cat#1 721 034, 1:5000).

### MTT assay

Cells were seeded into 96‐well plates at a density of 5000 cells per well, and the cell growth rate was assessed by MTT assay (#M2128, Sigma‐Aldrich, Germany). Following a previously established protocol, the optical density (OD) of the different groups was measured at 595 nm at specific time points.^[^ ^62^ ^]^

### Tumor Sphere Formation Assay

Cancer cells were placed in a 96‐well Clear Round Bottom Ultra‐Low Attachment Microplate (#7007, Corning, USA). Cells were seeded into plates with covalently bonded hydrogels to minimize cell attachment and facilitate the formation of tumor spheres. These spheres were photographed using a Carl Zeiss microscope (10 × objective, Axio Observer, Germany). Subsequently, ImageJ software was used to measure the sizes of the spheres.

### Colony Formation Assay

Cells were seeded in 6‐well plates and cultured in medium supplemented with 10% FBS for the indicated periods. Cells were washed three times with PBS, fixed with paraformaldehyde (PFA; #158127, Sigma‒Aldrich, Germany) for 15 min, and stained with 0.5% crystal violet (#C6158, Sigma‐Aldrich, Germany) for 15 min. After staining, the plates were rinsed with gently running water. The colonies were photographed, and they were counted using ImageJ.

### Transwell Migration and Invasion Assays

Cells in 100 µL of serum‐free medium were seeded into the upper chamber of a transwell insert (#3422, Corning, USA) for migration and invasion assays, and 700 µL of normal medium supplemented with 10% FBS was added to the lower chamber. For the migration assay, cells that crossed the transwell membrane were observed after 16 h of incubation. The cells were fixed with 4% paraformaldehyde for 15 min and stained with 0.5% crystal violet (#C6158, Sigma‒Aldrich, Germany) for 10 min. Subsequently, the membrane was carefully excised from the chamber and mounted on a glass slide using DPX mounting medium (#06522, Sigma‒Aldrich, Germany), and images were captured via microscopy (M165 FC, Leica, Germany). For the invasion assay, the upper chamber was precoated with 100 µL of Matrigel (#356 230, Corning, USA) at 37 °C for 2 to 3 h before cell seeding. Cells crossed the transwell membrane were observed after 24 h of incubation. The remaining steps were identical to those of the migration assay.

### Western Blotting Assay

Cells were harvested, washed once with phosphate‐buffered saline (PBS), and lysed using RIPA lysis buffer (150 mM NaCl, 50 mM Tris‐HCl, 0.5% SDS, and 1% Triton X‐100) supplemented with protease inhibitor (#P8340, Sigma‒Aldrich, Germany) and phosphatase inhibitor cocktails (#P0044, #P5726, Sigma‒Aldrich, Germany). The protein concentration was determined by using the Bio‐Rad protein assay kit (#5000006, Bio‐Rad, USA). Protein samples, along with protein standards, were loaded onto SDS‐PAGE gels and separated. Subsequently, proteins were transferred onto a nitrocellulose membrane (NC; #1620112, Bio‐Rad, USA) using a transfer apparatus at a constant voltage. The NC membrane was then blocked with nonfat dry milk, followed by overnight incubation with primary antibodies at 4 °C. The membrane was incubated with HRP‐conjugated secondary antibodies for 1 h at room temperature. Details regarding the primary and secondary antibodies used can be found in antibodies of the experimental section. Finally, the membranes were incubated with Clarity Western ECL Substrate (#1705061, Bio‐Rad, USA) for visualization using an imaging system (Bio‐Rad, USA).

### Co‐Immunoprecipitation

To investigate the interaction between EGFR and HER3, cells were lysed using NP‐40 buffer, which contained 20 mM Tris HCl, 137 mM NaCl, 10% glycerol, 1% NP‐40, and 2 mM EDTA, along with a protease inhibitor cocktail to prevent protein degradation. The total cell lysates were incubated overnight at 4 °C with anti‐HER3 antibodies or rabbit IgG as a negative control. The protein‐antibody complexes were then pulled down using protein A/G agarose beads (#20423, Thermo Fisher Scientific, USA) for 4 h at 4 °C. Following extensive washing to remove nonspecific binding, the immunoprecipitated proteins were analyzed by Western blotting to assess EGFR‐HER3 interaction.

### Enzyme Linked Immunosorbent Assay

The human PCSK9 Simple Step ELISA Kit (ab209884; Abcam) was used to quantify the secreted PCSK9 levels in serum samples from the mouse orthotopic model. Mice were euthanized, and blood was collected via cardiac puncture. The blood was then transferred to a serum separation tube with an EDTA coating and centrifuged at 3800 rpm for 10 min to separate the serum. The serum was diluted with Sample Diluent NS. A standard curve was prepared using lyophilized recombinant human PCSK9 protein, which was initially diluted to a concentration of 400 ng mL^−1^, and subsequent dilutions were made according to the manufacturer's instructions. For the ELISA procedure, 50 µL of the Antibody Cocktail was added to each well of the 96‐well plate and incubated for 1 h at room temperature on a plate shaker set to 400 rpm. After washing each well three times with 350 µL of Wash Buffer PT, 100 µL of TMB Development Solution was added to each well, and the plate was incubated in the dark for 10 min, with continuous shaking at 400 rpm. Finally, 100 µL of Stop Solution was added to each well to halt the reaction. The plate was shaken for 1 min to ensure proper mixing, and the optical density (OD) at 450 nm was recorded to quantify PCSK9 levels in the serum.

### Immunofluorescence Staining

The coverslips were sterilized with ultraviolet light prior to use. Cells were seeded onto coverslips in 6‐well plates, washed three times with PBS, fixed with 4% PFA for 10 min, and permeabilized with 0.2% Triton X‐100 (#T8787, Sigma‒Aldrich, Germany) for 10 min. Cells were blocked with 3% bovine serum albumin (BSA, Catalog number) and incubated overnight with primary antibodies at 4 °C. The cells were incubated with Alexa Fluor–conjugated secondary antibodies for 1 h at room temperature. The cells were counterstained with Hoechst 33 342 (#H3570, Thermo Fisher Scientific, USA), and mounted onto glass slides for visualization by using a confocal microscope (Carl Zeiss Confocal LSM710, Germany). Antibodies used in immunofluorescence staining are the Materials and Methods experimental section. Intensity profiles of line scan were conducted in Carl Zeiss software using the Profile function and figures of intensity profile were performed using GraphPad Prism 9.0 software. Colocalization with ratio of CD44 was performed using Image J software.

### Immunohistochemistry

Immunohistochemical analysis was performed on tissue microarray slides (ID: F551101, Shanghai Outdo Biotech Company, China) with samples from patients with TNBC (characterized by negative expression of ER, PR, and HER2). The acquisition of human tissue microarrays was approved by the Ethics Committee of Shanghai Outdo Biotech Company (Approval No.: SHXC2021YF01). IHC staining was performed utilizing the Mouse and Rabbit Specific HRP/DAB (ABC) Detection IHC Kit (ab64264, Abcam, UK) following the manufacturer's protocol. IHC scores were calculated based on the percentage of positively stained cells, with the following criteria: 0 for no staining, 1 for less than 1% of positively stained cells, 2 for 1% to 10%, 3 for 11% to 33%, 4 for 34% to 66%, and 5 for 67% to 100%. Antibodies used in IHC staining are the Materials and Methods experimental section.

### Hematoxylin & Eosin Staining

Samples from the left lungs and orthotopic tumors were initially fixed with 4% PFA and dehydrated for ≈1 week. Following dehydration, the samples were rinsed with gently running water and subsequently embedded in paraffin. The paraffin‐embedded samples were then sectioned into 3‐µm‐thick tissue sections. These sections were deparaffinized and stained with H&E.

### Gene Knockdown and Overexpression

All shRNA and overexpression plasmids were procured from Vector‐Builder Company (Chicago, USA), with specific target sequences detailed in Tables S2 and S3, Supporting Information. The sequences of the shRNAs were determined by calculating the H‐b indexes of the candidates to ensure optimal knockdown efficiencies.^[^ ^63^, ^64^ ^]^ The efficiencies of knockdown or overexpression were evaluated by using qPCR and Western blotting.

### Lipid Extraction

Adherent cells (1 × 10^7^) were trypsinized and centrifuged at 1000 rpm for 3 min at 4 °C. Following this, 1 mL of chloroform: methanol: water (2:1:1, v/v/v) was added to the cell pellet. The sample was vortexed and incubated on ice for 10 min to ensure proper mixing. After incubation, the sample was centrifuged at 8000 rpm for 10 min. The lower organic phase was carefully collected, and the upper phase was re‐extracted by adding 1 mL of chloroform: methanol: water (2:1:1). This mixture was vortexed and centrifuged again at 8000 rpm for 10 min, and the lower organic phase was collected. Finally, the lower organic phase was dried under vacuum at 40 °C using a speed‐vac concentrator (Savant SPD121P, Thermo Scientific).

### Cholesterol Detection by High‐Performance Liquid Chromatography

The chromatographic separation of cholesterol was performed using a mobile phase consisting of isopropanol/acetonitrile/water (60:30:10, v/v/v) at a flow rate of 1 mL mi^−1^n. The column temperature was maintained at 28 °C, and the detection wavelength was set at 205 nm. Cholesterol identification was based on its retention time (RT) and the full UV spectrum. Cholesterol exhibited a retention time of ≈9.754 min. To quantify cholesterol levels, the area under the curve (AUC) was calculated from the chromatographic data.

### Cholesterol Detection using Cholesterol Test Kit or Filipin III

Total cholesterol levels in lipid samples were measured using a cholesterol test kit (CHOD‐PAP) (A030, Huili‐Changchun, China). A 1:10 mixture of the enzyme reagent and assay buffer, preheated to 37 °C, was added to the lipid samples. The mixture was vortexed thoroughly and centrifuged at 8000 rpm for 2 min. The upper phase was carefully transferred to a 96‐well plate. The plate was incubated at 37 °C for 15 min, and the optical density (OD) of the samples was then measured at 540–570 nm to quantify the cholesterol content. For visualization of cholesterol distribution in cells, Filipin III staining (SAE0087, Sigma‒Aldrich, Germany) was employed. Cells were seeded onto coverslips and fixed with 4% paraformaldehyde (PFA) for 10 min. After washing with phosphate‐buffered saline (PBS), the cells were blocked with 3% bovine serum albumin (BSA) and stained with 50 µg mL^−1^ Filipin III in the dark for 3 h at room temperature. Following staining, the coverslips were mounted onto glass slides for visualization under a confocal microscope to assess both intracellular and membrane cholesterol localization.

### Lipid Raft Detection

The Vybrant Alexa Fluor 555 Lipid Raft Labeling Kit (V‐34404, Invitrogen) was used to fluorescently label lipid rafts in live cells, with following the manufacturer's protocol: cells were trypsinized gently resuspended in CT‐B conjugate, and incubated for 10 min at 4 °C. Subsequently, the cells were washed three times with PBS, gently resuspended in anti‐CT‐B antibody, and incubated for 15 min at 4 °C. Cells were counterstained with Hoechst 33342. Finally, the coverslips were mounted onto glass microscope slides for visualization using a confocal microscope (Carl Zeiss Confocal LSM710, Germany). The results were quantified via ImageJ.

### Uptake of DiI‐LDL

Human DiI‐low density lipoprotein (human DiI‐LDL) (20614ES76, Yeasen) was diluted to 20 µg mL^−1^ in normal culture medium and added into the cells seeded onto glass coverslips in 6‐well plates. The plates were then incubated at 37 °C for 4 h. Cells were washed three times with PBS, fixed with 4% PFA for 10 min, and stained with Hoechst 33 342. The coverslips were mounted onto glass slides for visualization using a Carl Zeiss microscope (20 × objective, Axio Observer, Germany).

### Lung Metastasis Assay

All animal experiments performed in this study were approved by the University of Macau Animal Ethics Committee, with the approved protocol IDs (UMARE‐025‐2017 and UMARE‐026‐2017). Female nude mice aged 6 to 8 weeks were utilized for the experiments. A total of 1 × 10^6^ cells were injected into the tail vein of each mouse. Subsequently, the mice were euthanized 1 day, 1 week, 2 weeks, 4 weeks, and 5 weeks postinjection, and the formation of metastatic tumors were examined using an Olympus fluorescence microscope (MVX10, Japan), as described previously.^[^ ^31^ ^]^

### Orthotopic Xenografts

For the orthotopic xenograft, female NOD/SCID mice aged 6 to 8 weeks were utilized. A total of 1 × 10^6^ cells were inoculated into the fifth fat pad of each mouse. Subsequently, tumor size and mouse weight were recorded every week. At 6 weeks post inoculation, the mice were euthanized. Metastatic tumors were assessed by imaging tissues from the bone, iliac lymph node (LN), lung, renal LN, mediastinal LN, intestine, liver, heart, and brain, using an Olympus fluorescence microscope. The orthotopic tumor volume was calculated as: length × (width)^2^/2. Metastatic colonies on the left lungs and lymph nodes were counted to evaluate metastatic ability of cells in vivo, as described previously.^[^ ^61^ ^]^

### Statistical Analysis

The results are presented as the means ± standard deviation (SD), unless otherwise indicated. Statistical comparisons and differences were assessed using *t‐*test or ANOVA. Significance was determined at *p*‐values of *p < 0.05, **p < 0.01, *** *p* < 0.001, ****p < 0.0001 and ns, not significant. All statistical analyses were performed using GraphPad Prism 9.0 software.

### Clinical Database Availability

The following database websites were used to check clinical correlation of PCSK9 and disease progression: UALCAN (https://ualcan.path.uab.edu/index.html), cBioPortal database (https://www.cbioportal.org/),^[^ ^33^ ^]^ Kaplan–Meier Plotter (https://kmplot.com/analysis/) and ARCHS4 platform (https://maayanlab.cloud/archs4/). Broad Institute Single Cell Portal (https://singlecell.broadinstitute.org/single_cell)

## Conflict of Interest

The authors declare no conflict of interest.

## Author Contributions

T.H.L and K.Q.L. conceived the study and designed the experiments. T.H.L. conducted the experiments. R.F.W provided the 231‐GFP and 4–11 cell lines and helped with some of the experiments. T.H.L and K.Q.L analyzed the data and wrote the manuscript.

## Supporting information



Supporting Information

## Data Availability

The data that support the findings of this study are available from the corresponding author upon reasonable request.
