# Supplementary material for: PCSK9 Promotes the Malignancy of Triple‐negative Breast Cancer Cells by Reducing Cholesterol Levels at the Plasma Membrane to Activate EGFR and HER3
Source: Adv Sci (Weinh). 2025 Apr 7;12(20):2408514. doi: 10.1002/advs.202408514 (PMC12120737; doi:10.1002/advs.202408514)
Supplement: Supplementary file 1 — Supporting Information [file ADVS-12-2408514-s001.docx]

**Supporting Information**

**PCSK9 Promotes the Malignancy of Triple-negative Breast Cancer Cells by Reducing Cholesterol Levels at the Plasma Membrane to Activate EGFR and HER3**

Tianhong Li^1^, Renfei Wu^1^, Kathy Qian Luo^1,2*^

^1^Department of Biomedical Sciences, Faculty of Health Sciences, University of Macau, Taipa, Macao SAR, 999078, China

^2^Ministry of Education Frontiers Science Center for Precision Oncology, University of Macau, Taipa, Macao SAR, 999078, China

*Corresponding author. Email: [kluo@um.edu.mo](mailto:kluo@um.edu.mo)

**Supplementary figures**


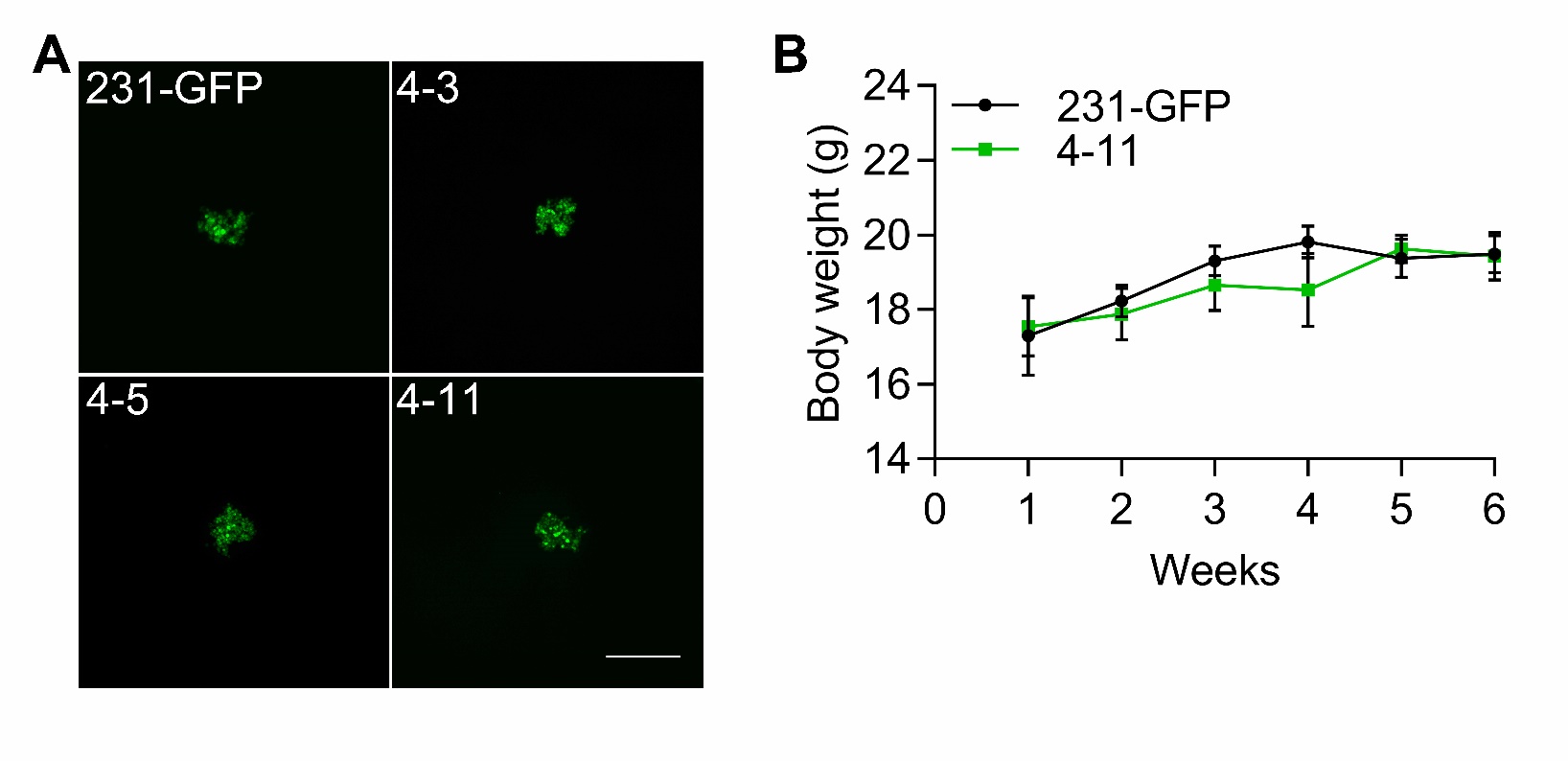


**Figure S1. The proliferative and metastatic abilities of 231-GFP derived cells.** (A) Representative fluorescence images of the tumor spheres formed by 231-GFP, 4-3, 4-5, and 4-11 cells on day 1. Cells were seeded onto round-bottom 96-well plates specifically designed with a hydrogel coating for ultralow attachment, creating conducive conditions for suspension culture. Scale bar, 200 µm. (B) Body weights of NOD/SCID mice bearing orthotopic tumors derived from 231-GFP and 4-11 cells.

**
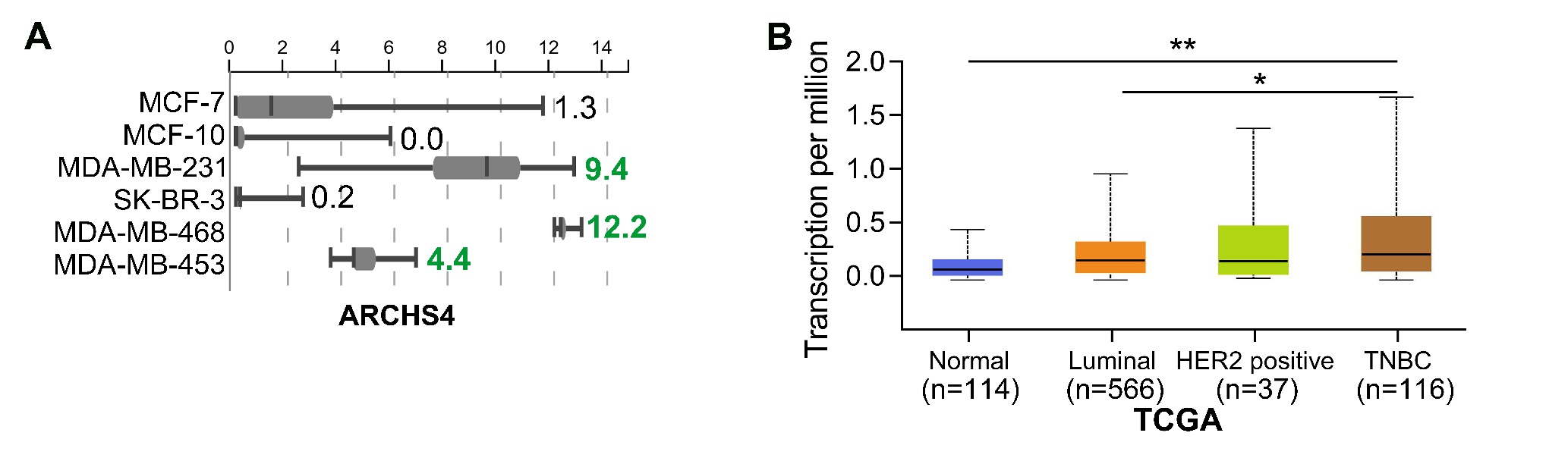
**

**Figure S2. PCSK9 expression is elevated in cell lines and clinical samples.** (A) Expression levels of PCSK9 in six breast cancer cell lines analyzed on the ARCHS4 platform. The green label represents three TNBC cell lines. (B) PCSK9 mRNA expression levels in different subtypes of breast cancer from TCGA dataset in the UALCAN platform. Sample numbers are shown for each subtype. The significance of differences was determined by one-way ANOVA (B)


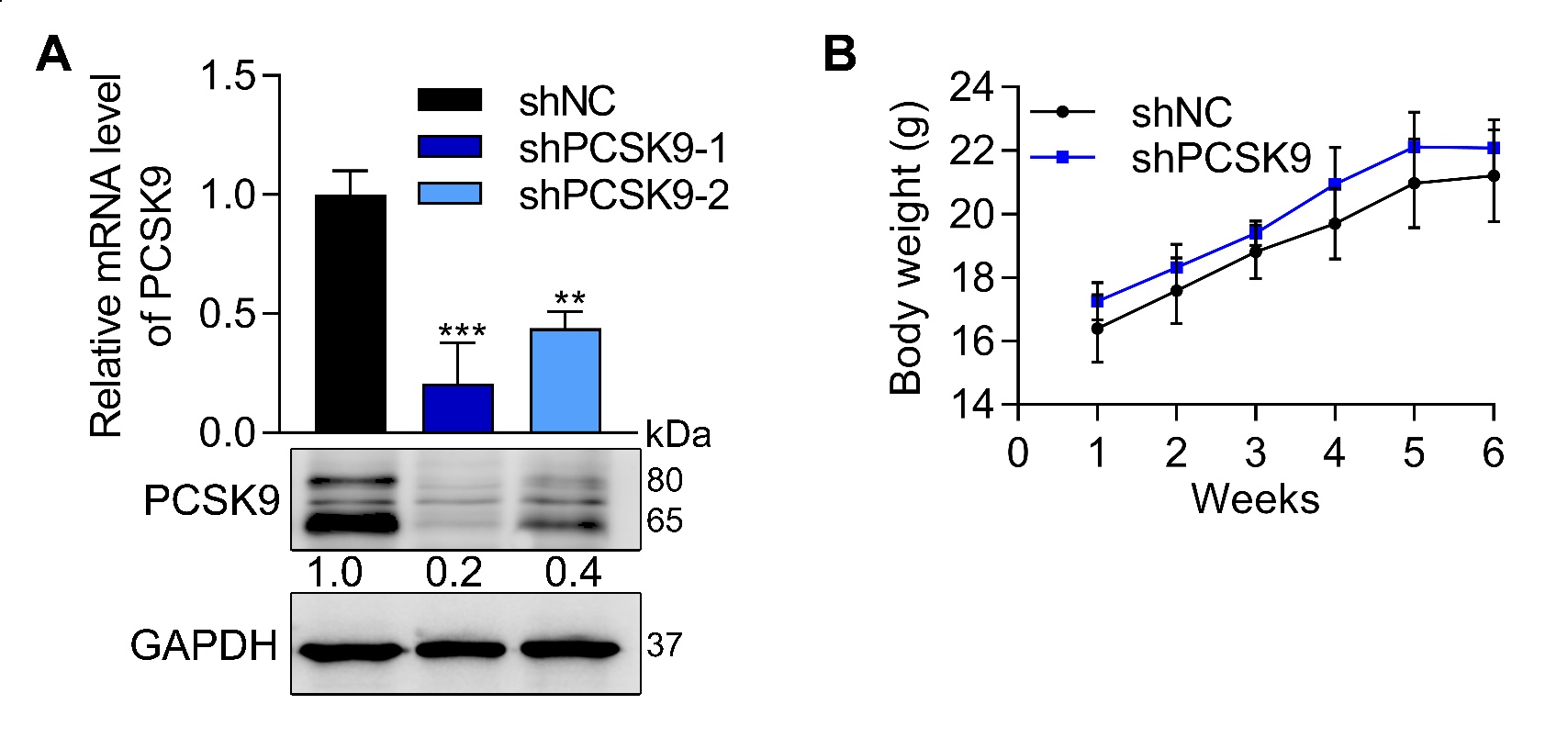


**Figure S3. Efficiency of shRNAs in reducing PCSK9 expression in 4-11 cells. (**A) Knockdown efficiencies of PCSK9 were detected at the mRNA level by qPCR (top) and protein levels by Western blotting (bottom). (B) Body weights of NOD/SCID mice bearing orthotopic tumors derived from 4-11-shNC and shPCSK9 cells. The significance of differences was determined by one-way ANOVA (A), or two-way ANOVA (B).


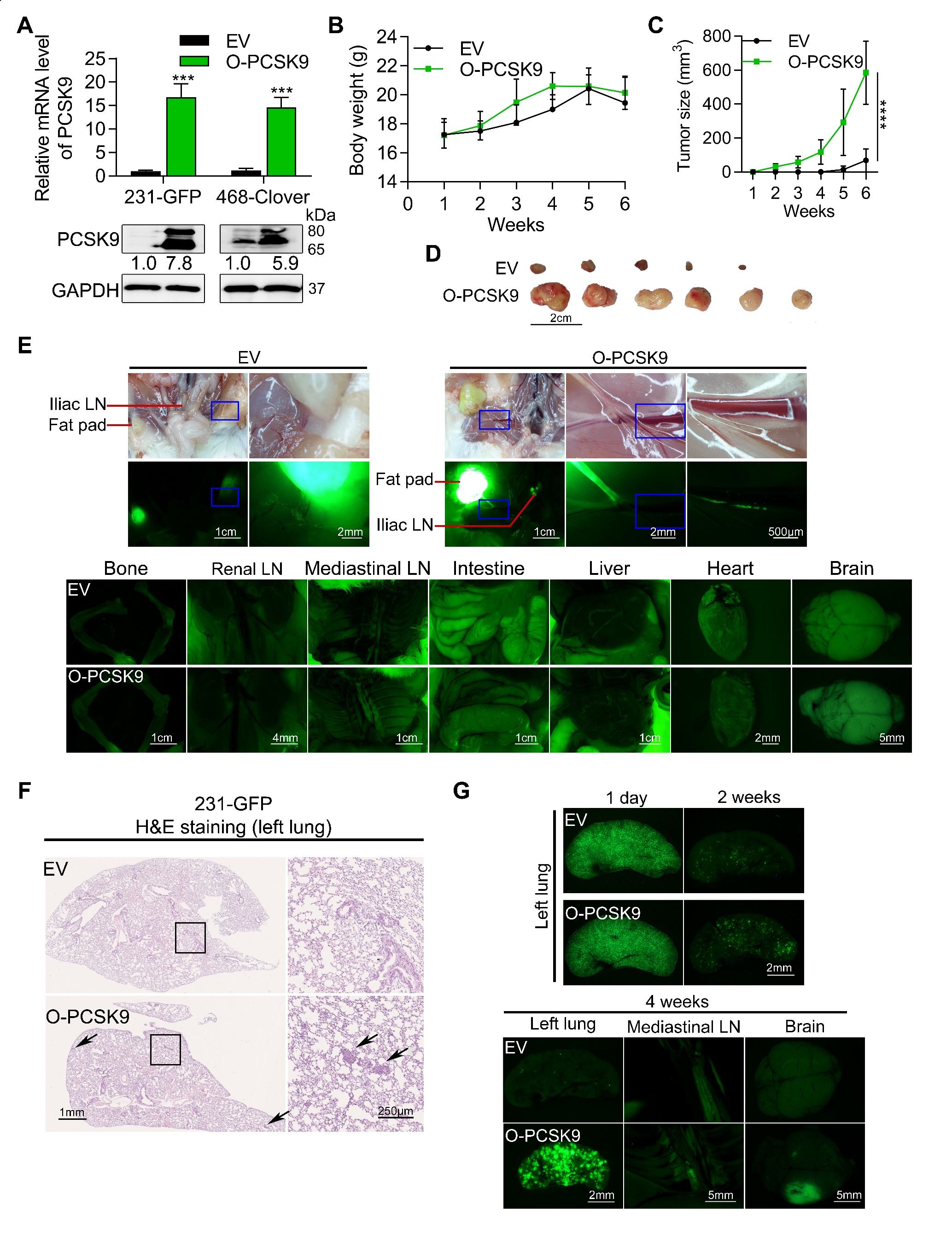


**Figure S4. Overexpression of PCSK9 increased metastasis of TNBC cells.** (A) Overexpression efficiencies of PCSK9 were assessed at both the mRNA level by qPCR (top) and protein levels by Western blotting (bottom) in 231-GFP and 468-Clover cells. (B) Body weights of NOD/SCID mice bearing orthotopic tumors derived from the 231-GFP-EV and 231-GFP-O-PCSK9 cells. (C) Primary tumor growth rate of 231-GFP cells with EV or O-PCSK9 (n=6−7). (D) Representative images of primary tumors after 6 weeks. (E) Representative images of primary and metastatic tumors in the examined organs or tissues including fad pad, bone, renal LN, mediastinal LN, intestine, liver, heart and brain. (F) H&E staining of the left lung with some metastatic colonies. (G) Representative images of left lung, mediastinal LN and brain post tail vein injections. The significance of differences was determined by two-way ANOVA (A, B, C).

**
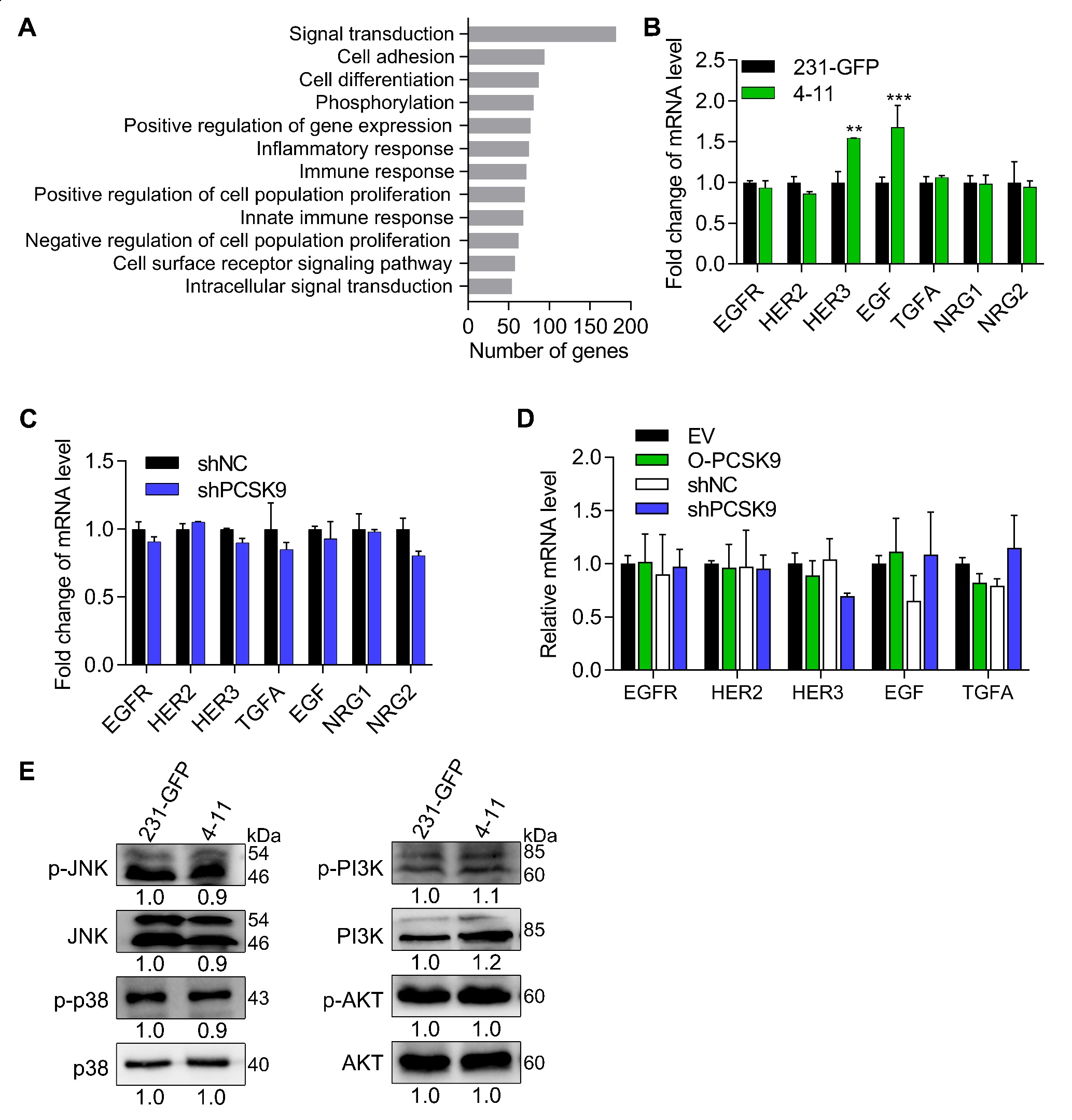
**

**Figure S5. PCSK9 did not affect the mRNA and protein levels of multiple molecules**. (A) Gene Ontology (GO) analysis revealed the enrichment of the cancer-related pathways from 894 genes in 4-11 cells compared to 231-GFP cells (*p* <0.05; fold change ≥2 or ≤0.5; FPKM of 231-GFP ≥1). (B) The RNA sequencing results revealing the relative mRNA levels of EGFR, HER2, HER3, TGFA, EGF, NRG1 and NRG2 between 4-11 and 231-GFP cells. (C) The RNA sequencing results of those genes between the shNC and shPCSK9 of 4-11 cells. 41 downregulated genes were found in shPCSK9 cells (*p* <0.05; fold change ≤0.5 and FPKM of shNC ≥1). (D) Relative mRNA levels of EGFR, HER2, HER3, EGF, and TGFA in the empty vector and PCSK9 overexpression group of 231-GFP cells, and in the shNC and shPCSK9 groups of 4-11 cells as measured using qPCR. (E) Western blots depicted the phosphorylated or total protein levels of JNK, p38, PI3K, and AKT in 231-GFP and 4-11 cells. The significance of differences was determined by two-way ANOVA (B, C, D).


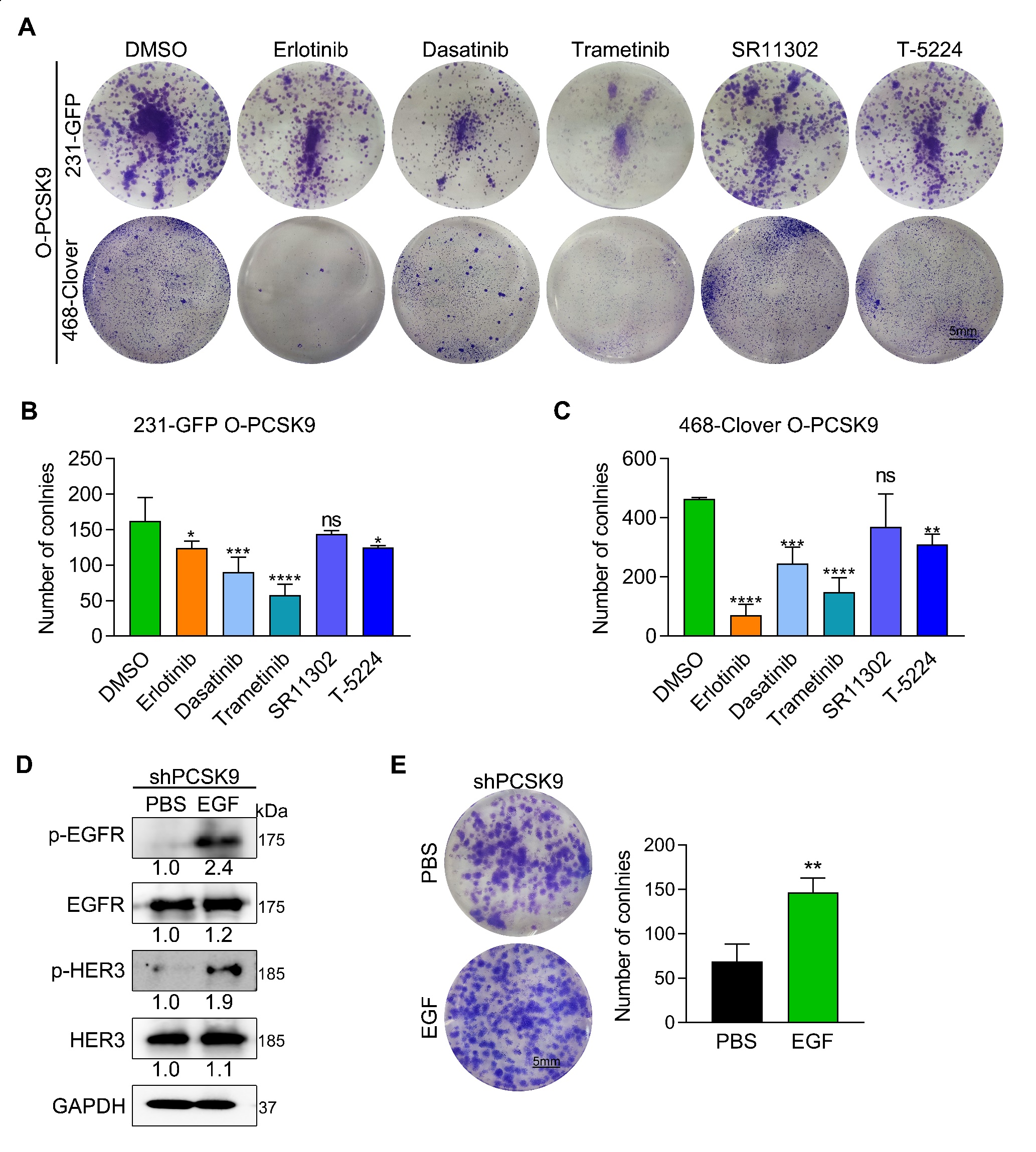


**Figure S6. Inhibitors of EGFR, MEK, Src and cJun, and activator of EGFR/HER3 affected PCSK9-promoted proliferation and metastasis in 231-GFP and 468-Clover cells.** (A, B, C) Representative images and quantified results of colony formation for PCSK9-overexpressing cells treated with the inhibitors of EGFR, MEK, Src and cJun. (D) Western blotting results showing the protein levels of EGFR and HER3 in shPCSK9 of 4-11 cells treated with EGF. (E) Representative images and quantified results of colony formation assay treated with EGF. The significance of differences was determined by one-way ANOVA (B, C) or Student’s *t-test* (E).


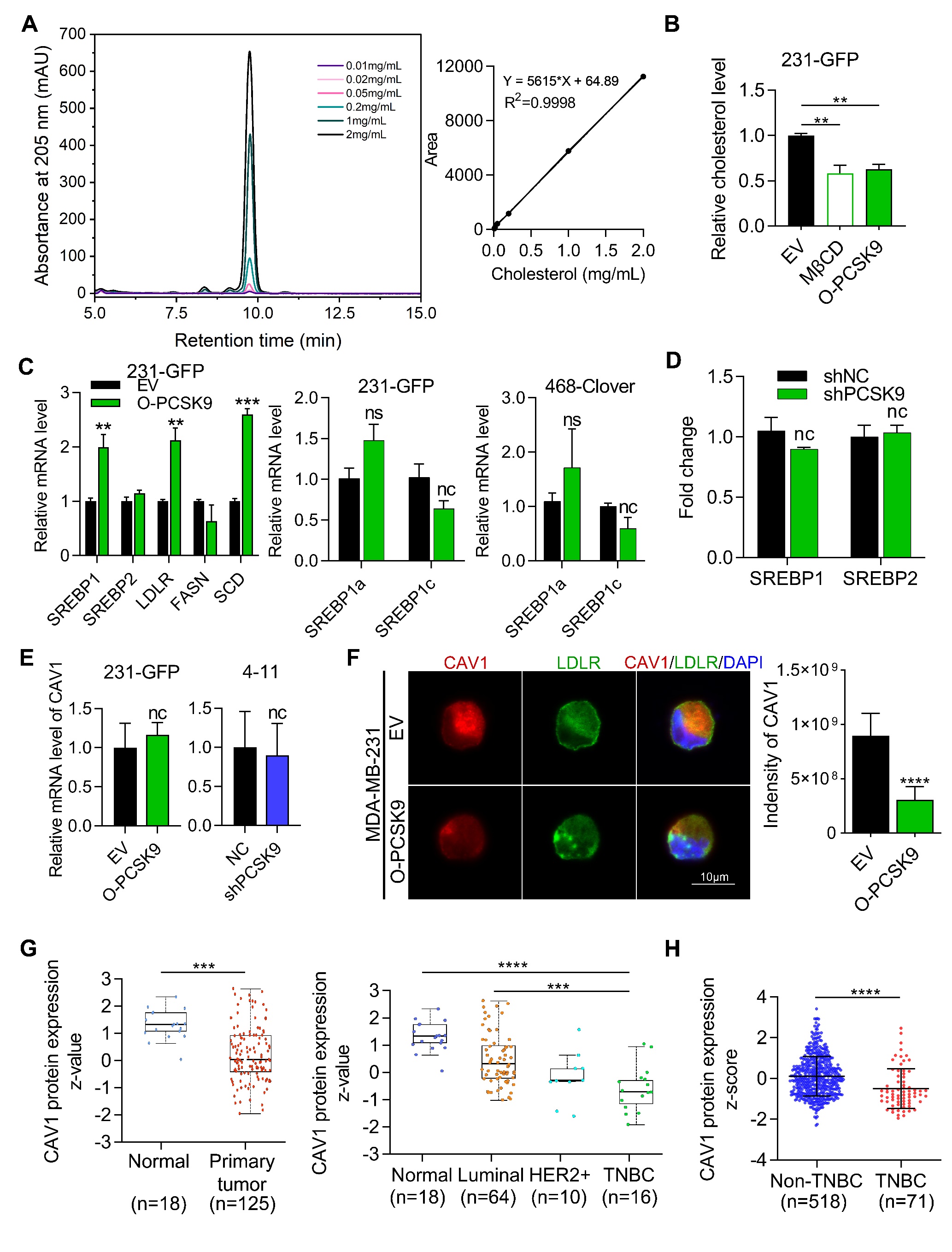


**Figure S7. PCSK9 activated EGFR and HER3 by reducing cholesterol levels in cell membranes**. (A) Left: the cholesterol peak shows a characteristic retention time (RT) of 9.75 minutes: right: the 6-point calibration curve used to quantify the amount of cholesterol by the area under the curve (AUC). (B) The dried lipids obtained after the methanol/chloroform purification were measured by using a cholesterol test kit (V-34404, Invitrogen). (C) Relative mRNA levels of SREBP1a, SREBP1c, SREBP1, SREBP2, LDLR, FASN, and SCD were measured by qPCR in 231-GFP cells and 468-Clover cells with EV or O-PCSK9. (D) RNA sequencing results showed relative mRNA levels of SREBP1 and SREBP2 in 4-11 cells with shNC or shPCSK9. (E) Relative mRNA levels of CAV1 determined by qPCR. (F) Co-immunostaining of LDLR and CAV1 in MDA-MB-231 cells with EV or O-PCSK9 and quantification of CAV1 intensity (n ≥ 30 cells). (G) The protein levels of CAV1 in normal controls and breast cancer patients, as well as in normal, luminal, HER2+, and TNBC patients from the UALCAN platform. (H) The protein levels of CAV1 in non-TNBC and TNBC patients from the cBioPortal platform. Significant differences were determined by one-way ANOVA (B, G), two-way ANOVA tests (C, D), or Student’s *t-test* (E, F, G, H)


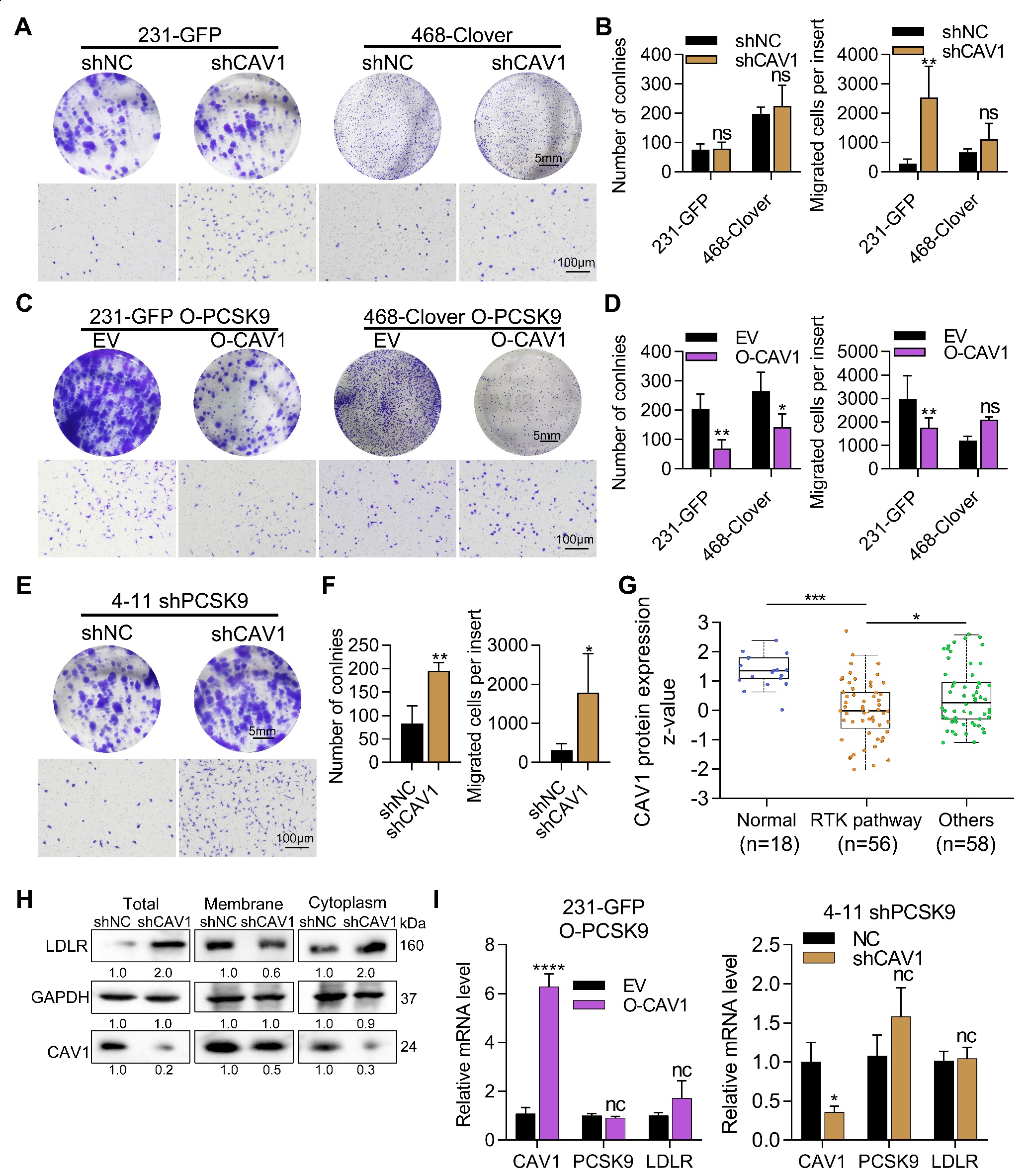


**Figure S8. Treatment with MβCD or overexpression of PCSK9 reduced cholesterol levels on the cell membrane.** (A and C) Representative images and quantified results of colony formation and transwell migration assays. (B and D) Representative images and quantified results of colony formation and transwell migration assays. (E and F) Representative images and quantified results of colony formation and transwell migration assays. (G) CAV1 protein expression profile based on RTK pathway status in breast cancer samples from the UALCAN platform. (H) LDLR and CAV1 protein level in knockdown CAV1 cells. (I) Relative mRNA levels of CAV1, PCSK9 and LDLR were measured by qPCR. Significant differences were determined by one-way ANOVA (G), two-way ANOVA tests (B, D, I), or Student’s *t-test* (F).

**
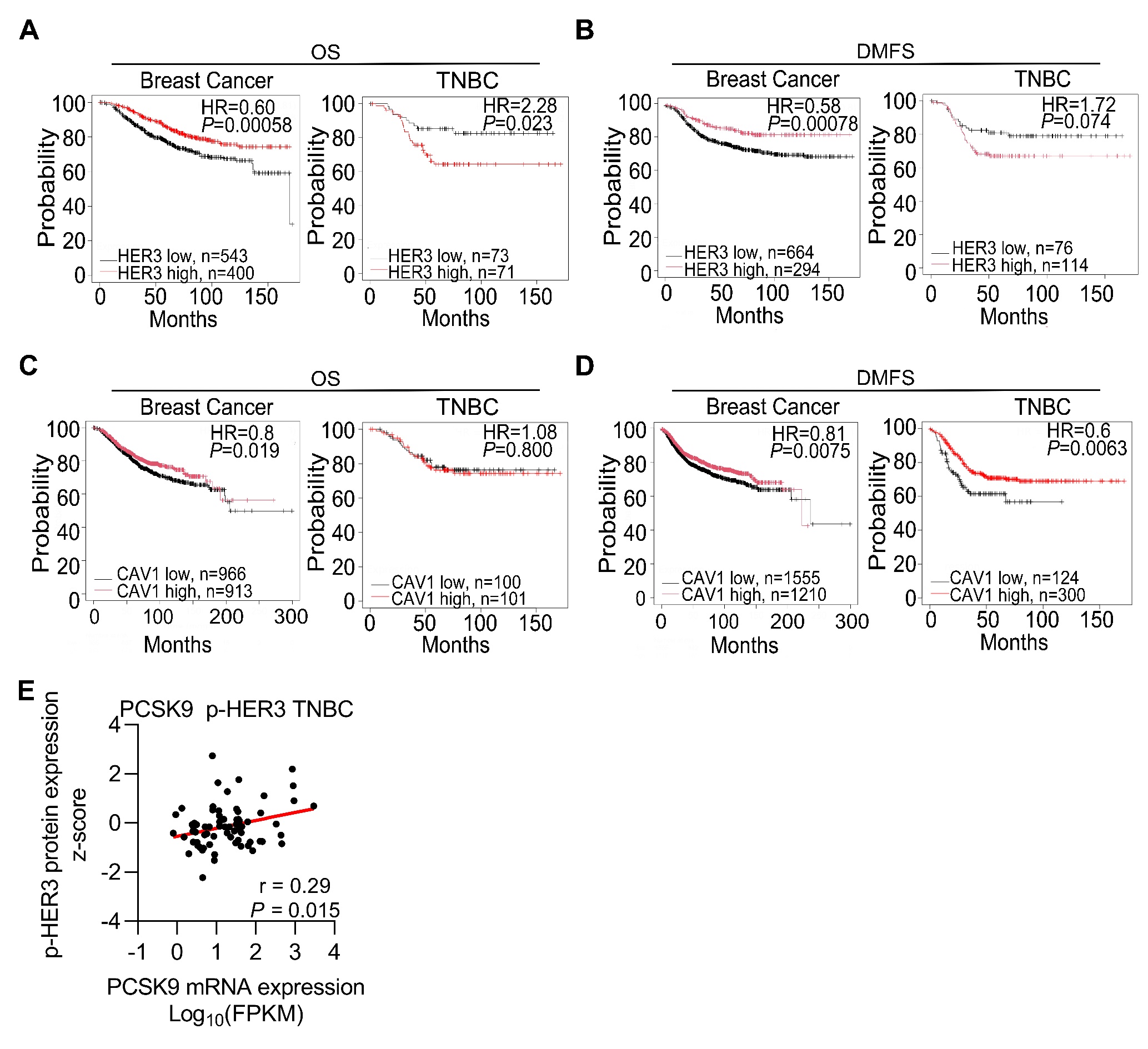
**

**Figure S9. High expression of HER3 and low expression of CAV1 are associated with poor clinical prognosis in TNBC.** (A) Kaplan–Meier plots of OS curves in breast cancer patients and TNBC patients stratified by HER3 expression. (B) Kaplan–Meier plots of DMFS curves in breast cancer patients and TNBC patients grouped based on HER3 expression. (C) Kaplan–Meier plots of OS curves in breast cancer patients and TNBC patients stratified by CAV1 expression. (D) Kaplan–Meier plots of DMFS curves in breast cancer patients and TNBC patients grouped based on CAV1 expression. (E) Positive correlation between the mRNA expression levels of PCSK9 and phosphorylated levels of HER3 protein in TNBC patients from the cBioPortal platform (n = 70).

**Supplementary tables**

**Table S1. List of primers for qPCR.**

| Gene | F (5’) | R (3’) |
| --- | --- | --- |
| GAPDH | GTCTCCTCTGACTTCAACAGCG | ACCACCCTGTTGCTGTAGCCAA |
| PCSK9 | ATGGTCACCGACTTCGAGAAT | GTGCCATGACTGTCACACTTG |
| SREBP1a | TTCCGAGGAACTTTTCGCCG | GGGAGGGCTTCCTGTAGAGA |
| SREBP1c | AGCCATGGATTGCACTTTCG | GGGAGGGCTTCCTGTAGAGA |
| SREBP1 | ACAGTGACTTCCCTGGCCTAT | GCATGGACGGGTACATCTTCAA |
| SREBP2 | CCTGGGAGACATCGACGAGAT | TGAATGACCGTTGCACTGAAG |
| SCD | TCTAGCTCCTATACCACCACCA | TCGTCTCCAACTTATCTCCTCC |
| FASN | AAGGACCTGTCTAGGTTTGATGC | TGGCTTCATAGGTGACTTCCA |
| LDLR | TCTGCAACATGGCTAGAGACT | TCCAAGCATTCGTTGGTCCC |
| EGFR | CCCACTCATGCTCTACAACCC | TCGCACTTCTTACACTTGCGG |
| HER2 | TGTGACTGCCTGTCCCTACAA | CCAGACCATAGCACACTCGG |
| HER3 | GGTGATGGGGAACCTTGAGAT | CTGTCACTTCTCGAATCCACTG |
| EGF | TGGATGTGCTTGATAAGCGG | ACCATGTCCTTTCCAGTGTGT |
| TGFA | AGGTCCGAAAACACTGTGAGT | AGCAAGCGGTTCTTCCCTTC |
| NRG1 | CGGTGTCCATGCCTTCCAT | GCGAGTTTCTTAACAGGCTCT |
| NRG2 | GTGTGCCGCTCGAAAGGAA | ACACTTCAGCGATTGCTTCTC |
| CAV1 | GCGACCCTAAACACCTCAAC | ATGCCGTCAAAACTGTGTGTC |

**Table S2. List of shRNAs from Vector-Builder.**

| shRNA | ID | Target sequence (5’) |
| --- | --- | --- |
| shNC | VB010000-0007mbh | CCTAAGGTTAAGTCGCCCTCG |
| shPCSK9-1 | VB201009-1101gwj | CGGGGATACCTCACCAAGATC |
| shPCSK9-2 | VB220316-1026puq | ACAGAGTGACCACCGGGAAAT |
| shLDLR | VB230626-1706gxt | GATGAAGTTGGCTGCGTTAAT |
| shCAV1 | VB240507-1058nkb | GCAGTTGTACCATGCATTAAG |

**Table S3. List of overexpression vectors from Vector-Builder.**

| Vector | ID | Sequence length of gene (bp) |
| --- | --- | --- |
| O-PCSK9 | VB211026-1372phn | 6382 |
| O-CAV1 | VB241008-1884bwz | 5195 |
